# Lonafarnib Protects Against Muscle Atrophy Induced by Dexamethasone

**DOI:** 10.1002/jcsm.13665

**Published:** 2024-12-17

**Authors:** Sanghoon Bae, Van‐Hieu Mai, Seyoung Mun, Dalong Dong, Kyudong Han, Sunghyouk Park, Jung Keun Hyun

**Affiliations:** ^1^ Department of Nanobiomedical Science & BK21 NBM Global Research Center for Regenerative Medicine Dankook University Cheonan Korea; ^2^ Natural Product Research Institute, College of Pharmacy Seoul National University Seoul Korea; ^3^ Department of Microbiology, College of Science & Technology Dankook University Cheonan Korea; ^4^ Smart Animal Bio Institute Dankook University Cheonan Korea; ^5^ Center for Bio‐Medical Core Facility Dankook University Cheonan Korea; ^6^ College of Science & Technology Dankook University Cheonan Korea; ^7^ Institute of Tissue Regeneration Engineering (ITREN) Dankook University Cheonan Korea; ^8^ Department of Rehabilitation Medicine, College of Medicine Dankook University Cheonan Korea; ^9^ Wiregene Co. Ltd. Chungju Korea

**Keywords:** ANGPLT4, dexamethasone, drug repositioning, lonafarnib, muscle atrophy, UCP3

## Abstract

**Background:**

Muscle atrophy, including glucocorticoid‐induced muscle wasting from treatments such as dexamethasone (DEX), results in significant reductions in muscle mass, strength and function. This study investigates the potential of lonafarnib, a farnesyltransferase inhibitor, to counteract DEX‐induced muscle atrophy by targeting key signalling pathways.

**Methods:**

We utilized in vitro models with C2C12 myotubes treated with DEX and in vivo models with *Caenorhabditis elegans* and DEX‐treated Sprague–Dawley rats. Myotube morphology was assessed by measuring area, fusion index and diameter. Muscle function was evaluated by grip strength and compound muscle action potential (CMAP) in the gastrocnemius (GC) and tibialis anterior (TA) muscles. Molecular mechanisms were explored through RNA sequencing and Western blotting to assess changes in mitochondrial function and muscle signalling pathways.

**Results:**

Lonafarnib (2 μM) significantly improved myotube area (1.49 ± 0.14 × 10^5^ μm^2^ vs. 1.03 ± 0.49 × 10^5^ μm^2^ in DEX, *p* < 0.05), fusion index (18.73 ± 1.23% vs. 13.3 ± 1.56% in DEX, *p* < 0.05) and myotube diameter (31.89 ± 0.89 μm vs. 21.56 ± 1.01 μm in DEX, *p* < 0.05) in C2C12 myotubes. In *C. elegans* , lonafarnib (100 μM) increased the pharyngeal pumping rate from 212 ± 7.21 contractions/min in controls to 308 ± 17.09 contractions/min at day 4 (*p* < 0.05), indicating enhanced neuromuscular function. In DEX‐induced atrophic rats, lonafarnib improved maximal grip strength (DEX: 13.91 ± 0.78 N vs. 1 μM lonafarnib: 16.18 ± 0.84 N and 5 μM lonafarnib: 16.71 ± 0.83 N, *p* < 0.05), increased muscle weight in GC, and enhanced CMAP amplitudes in both GC and TA muscles. Western blot analysis showed that lonafarnib treatment upregulated UCP3 and ANGPTL4 and increased phosphorylation of mTOR and S6 ribosomal protein (*p* < 0.05), indicating enhanced mitochondrial function and protein synthesis. Knockdown models further demonstrated that lonafarnib could partially rescue muscle atrophy phenotypes, indicating its action through multiple molecular pathways.

**Conclusions:**

Lonafarnib mitigates dexamethasone‐induced muscle atrophy by enhancing mitochondrial function and activating anabolic pathways. These findings support further investigation of lonafarnib as a therapeutic agent for muscle atrophy in clinical settings.

## 1 Introduction

Muscle atrophy, including sarcopenia and glucocorticoid‐induced muscle wasting, is characterized by the loss of muscle mass, strength and function and presents significant public health concerns [1]. Sarcopenia is typically observed in the elderly population, while glucocorticoid‐induced muscle atrophy can result from treatments such as dexamethasone. Both conditions are associated with an increased risk of falls, functional decline, hospitalizations and mortality [S1]. Various aetiologies contribute to the development of sarcopenia, including hormonal changes, satellite cell decrements and chronic inflammation [S2], while glucocorticoid‐induced atrophy is driven by increased protein degradation and decreased protein synthesis mediated by pathways such as the ubiquitin‐proteasome system and autophagy [S3, S4]. Despite extensive research, finding effective pharmacological treatments remains a challenge. Several treatments that have been explored, including hormonal therapies [S5, S6], anabolic agents [S7] and amino acid supplementation [S8], have been limited by side effects or inadequate efficacy [S7–S9]. Myostatin inhibitors, while showing early promise, have not yielded significant benefits in human trials [2]. The complexity of muscle atrophy as a multifactorial condition requires novel, safe and effective long‐term therapeutic strategies.

Lonafarnib, a farnesyltransferase inhibitor, was originally developed as an investigational drug for cancer treatment [S10]. It was recently approved by the FDA for improving survival rates in patients with Hutchinson–Gilford progeria syndrome (HGPS) and progeroid laminopathies, rare genetic disorders causing premature aging [3]. Experimental studies also showed positive findings, such as the prevention of nuclear blebbing [S11, S12]. Lonafarnib has also been shown to be effective for treating other diseases, including hepatitis delta virus infection [S13] and neurodegenerative diseases such as tauopathy [S14].

In previous studies, increased farnesyltransferase expression and farnesylated protein levels were shown to induce metabolic dysfunction and inflammation in burn‐induced skeletal muscle, and a farnesyltransferase inhibitor was shown to prevent muscle damage [4, S15]. The phosphatidylinositol 3‐kinase (PI3K)/AKT/mammalian target of rapamycin (mTOR) signalling pathway is involved in regulating protein synthesis and cell growth in skeletal muscle [5, S16]. The Ras signalling pathway can influence the PI3K/Akt/mTOR pathway, thereby affecting muscle protein synthesis and degradation [6, S17]. The inhibition of the Ras signalling pathway could help reduce muscle atrophy by promoting protein synthesis and inhibiting protein degradation [S18]. Another pathway influenced by Ras signalling is the MAPK/ERK pathway, which is involved in cell proliferation, differentiation, and survival [7, S19]. Modulation of these pathways could also potentially have an impact on muscle atrophy, including conditions induced by glucocorticoids. We hypothesized that lonafarnib could reverse dexamethasone‐induced muscle atrophy and restore muscle health to near‐normal levels. To test this, we used both in vitro and in vivo models to evaluate lonafarnib's effects on muscle mass, strength and function, as well as the underlying mechanisms. The study design included multiple groups to assess lonafarnib's therapeutic potential under various conditions of muscle atrophy.

The aim of this study was to explore the effects and potential mechanisms of lonafarnib on the recovery of dexamethasone‐induced atrophied skeletal muscles by utilizing both in vitro and in vivo models to provide a comprehensive understanding of its therapeutic potential in treating muscle atrophy.

## 2 Methods

### 2.1 Reagents and Antibodies

Lonafarnib was purchased from Cayman Chemical Company (Ann Arbor, MI, USA) and was dissolved in dimethyl sulfoxide (DMSO). Dulbecco's modified Eagle's medium (DMEM) was purchased from Welgene (Gyeongsan, Korea). Penicillin–streptomycin (10 000 U/mL penicillin and 10 000 μg/mL streptomycin) was purchased from Sigma–Aldrich (St. Louis, MO, USA). Fetal bovine serum and horse serum were purchased from Corning Life Science (Glendale, AZ, USA). Unless otherwise specified, all other chemicals were obtained from Sigma–Aldrich. All other primary antibodies were purchased from Cell Signalling Technology (Danvers, MA, USA), DSHB (Iowa City, IA, USA), Santa Cruz Biotechnology (Santa Cruz, CA, USA), Abcam (Cambridge, MA, USA) and Invitrogen (Carlsbad, CA, USA) (Table S1), and the secondary antibodies were purchased from Jackson ImmunoResearch (West Grove, PA, USA) and Invitrogen.

### 2.2 C2C12 Cell Culture, Differentiation and Analysis

Myoblasts (C2C12) were purchased from ATCC (Manassas, VA, USA) and maintained in growth media (GM, DMEM supplemented with 10% fetal bovine serum and 1% P/S). To initiate differentiation, the cells were grown to 60%–70% confluence and incubated with differentiation media (DM, DMEM with 2% heat‐inactivated normal horse serum and 1% P/S) for 48 h. Then, fully differentiated myotubes were treated with 150 μM dexamethasone (DEX) or lonafarnib (Lona) for 24 h. After treatment, myotubes were fixed with 4% paraformaldehyde and permeabilized with 0.1% Triton X‐100. Cells were blocked with 2% normal goat serum for 1 h and then incubated with primary antibodies overnight at 4°C. Antibodies MYH (myosin heavy chain 3) and DAPI (4′,6‐diamidino‐2‐phenylindole, blue) were used to stain myotube and nuclei, respectively. Detailed information on the antibodies is provided in Table S1. After primary antibody incubation, cells were washed and incubated with appropriate secondary antibodies conjugated to Alexa Fluor 488 dye (green).

**FIGURE 1:**
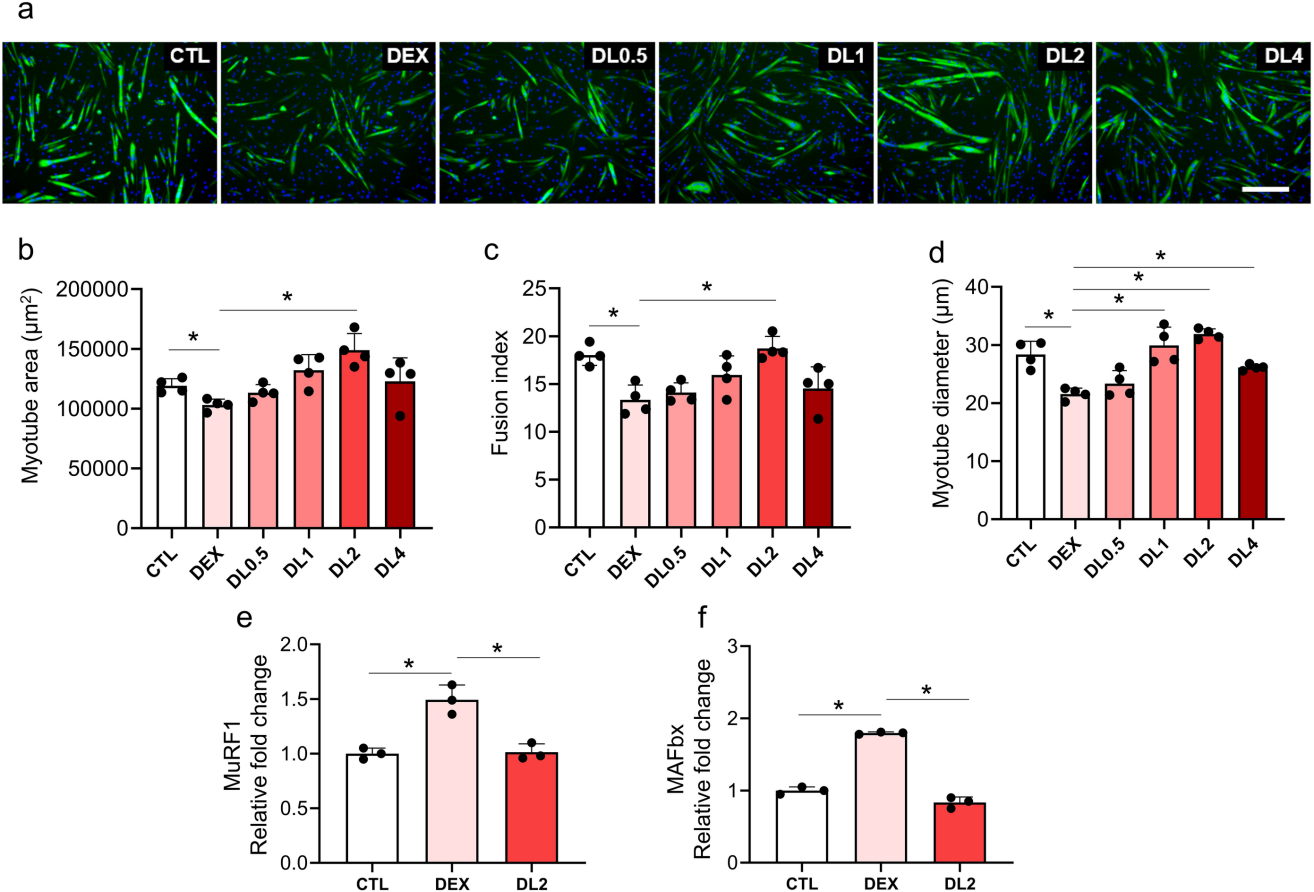
Effect of lonafarnib on dexamethasone‐induced muscle atrophy in in vitro models. (a) Representative microscopy images stained with MYH (green) and DAPI (blue) illustrating the morphological differences between the control group (CTL), dexamethasone‐induced muscle atrophy model (DEX), and DEX models treated with 0.5, 1, 2 or 4 μM lonafarnib (DL0.5, DL1, DL2 and DL4, respectively). (b) Quantitative analysis of myotube area. (c) Evaluation of the fusion index, d. Myotube diameter measurements. (e, f) Relative fold changes in MuRF1 (e) and MAFbx (f) gene expression levels determined by real‐time PCR analysis for each group. *p < 0.05 by one‐way ANOVA with the Games–Howell post hoc test.

The analysis of myotube area and fusion index was conducted by capturing and analysing five images per culture well. Fields of view were randomly selected across the entire well and centred to avoid overlap. Four culture wells were used per experimental group. Data shown are from a single experiment. The dots in Figure 1 represent the average of each well, with four dots representing the average of four individual wells. The myotube area and fusion index were calculated using the Myocount program as previously described [S20]. The calculation of fusion index is based on the number of nuclei in mature myotubes (myotubes with three or more fused nuclei) divided by the total number of nuclei multiplied by 100.

$$\text{Fusion index}\;\left(\mathrm{FI}\right)=\frac{\text{Number of nuclei in myotubes}}{\text{Total number of nuclei}}\times 100$$

The analysis of myotube diameter was conducted in 96‐well plates, with a total of four wells per condition. For each well, two fields of view were imaged, and for each field, three myotubes were measured. Each myotube diameter was measured at three different points to obtain an average. Only mature myotubes with three or more nuclei were selected for measurement, and radially growing myotubes were excluded.

### 2.3 Small Interfering RNA (siRNA) Transfection

C2C12 cells (1.3 × 10^5^ cells) were seeded in a 6‐well culture plate and cultured for 48 h in GM. To initiate differentiation, the cells were incubated with DM for 48 h. siRNA–transfection reagent complexes were prepared in serum‐free and antibiotic‐free media and incubated with cells for 4 h at 37°C in a CO_2_ incubator. Then, the media was replaced with fresh complete DM without antibiotics. After 24 h, the DM‐containing lonafarnib was changed. siRNA duplexes and transfection reagents were purchased from Bioneer (Daejon, Korea) and AptaBio (Yongin, Korea).

### 2.4 *Caenorhabditis elegans* Model

The *C. elegans* strain N2 (Bristol) was obtained from the Caenorhabditis Genetics Center (CGC, University of Minnesota, MN, USA) and cultivated following the standard protocol with *E. coli* OP50 as the feed source [8]. This model was selected due to its relevance in studying age‐related muscle atrophy, particularly through the measurement of pharyngeal pumping rate as an indicator of muscular decline [S21]. The bleaching method was used to obtain worm eggs for L1 larvae synchronization.

### 2.5 Pharyngeal Pumping Assay

Lonafarnib was diluted in NGM agar to different 10‐fold final concentrations (0, 1, 10 and 100 μM; equal to the adjusted DMSO concentration) in 6‐well plates. OP50 (200 μL) suspended in LB medium (OD_600 nm_ = 2.0) was spread to create a food lawn and air‐dried. Approximately 100 synchronized L1 nematodes were seeded on the above‐prepared plates and grown in a 20°C incubator. After 60 h of cultivation, the worms in the vehicle plate approached the L4 stage with tapered tails and vulva, after which the pharyngeal pumping assay started (day 0 on the graph timepoint) as previously described [S22]. For microscopic observation, an NGM layer with an OP50 lawn (OD_600 nm_ = 2.0) was cut into equal frames (2 × 2 cm^2^) and placed on microscope glass slides. At least three random worms from each drug concentration plate were manually picked, transferred to a prepared agar frame with a platinum wire, and then incubated for 30 min at 20°C before observation. At 20–40× magnification under a microscope (Olympus JP/BX50), the pharyngeal activity of *C. elegans* was recorded by a digital camera (AmScope MU1803) at 50 frames/s, after which pharyngeal pumping was tracked and scored via a video player at a twofold slower speed. The pharyngeal pumping rate was obtained by counting the cycle of grinder movements every 30 s repeated three times per single worm and then estimating the average per minute (ppm). The remaining nematodes in the culture plates were physically transferred to a new plate to avoid crossing with the descendent generation. The procedure was then repeated every following day.

### 2.6 Dexamethasone‐Induced in Vivo Model

All experimental protocols were approved by the animal study committee of Dankook University's Institutional Animal Care and Use Committee (approval No. 21‐006). Male S.D. rats (12 weeks old, 310–340 g) were housed at 23 ± 1°C under a 12‐h light/dark cycle. The rats had free access to food and water under controlled humidity (45%–50%) with air filtration. The rats were randomly divided into six groups (control group, CTL; CL1; CL5; dexamethasone group, DEX; DL1; and DL5) to assess the therapeutic and dose‐dependent effects of lonafarnib, and the rats in all groups were intraperitoneally (IP) injected. Rats in the CTL, CL1 and CL5 groups were injected with saline (10 mL/kg), while the other groups (DEX, DL1 and DL5) received dexamethasone (1 mL/kg; 0.25 mg/mL) from Day 1 to Day 6. Additionally, the lonafarnib‐treated groups (DL1 and DL5) were injected with lonafarnib (10 mL/kg) at concentrations of either 1 or 5 μM, respectively, during the same period. All injections were administered intraperitoneally for 6 days. Dexamethasone was administered daily at a dose of 1 mg/kg through intraperitoneal injection. The solubility of dexamethasone is 25 mg/mL, and it was dissolved in DMSO to achieve a final concentration of 0.25 mg/mL in a 5% DMSO solution. This concentration was selected to avoid precipitation of the dexamethasone. The total volume administered was 4 mL/kg, which corresponds to the chosen dose of 1 mg/kg of dexamethasone. This dosing protocol adheres to the IACUC guidelines, which recommend not exceeding a total daily injection volume of 20 mL/kg, including the volume for lonafarnib administration. Bodyweights were recorded every day. On day 7, all the rats were sacrificed, and the lower leg muscles were dissected. The tissue samples were quickly weighed and frozen in liquid nitrogen or embedded in O.C.T. compound (Sakura Finetek; Torrance, CA, USA). The atrophy rate was calculated as the ratio of muscle mass to body weight.

### 2.7 Grip Strength Measurement

Forelimb grip strength was measured by the rat pulling backward the bar attached to the grip strength meter (Jeung‐do B&P; Seoul, Korea); grip strength was measured every other day. The test was repeated three times, and the rest time was 15 min between tests. The maximal value among three measurements was used.

### 2.8 Electrophysiological Assessment

The rats were anaesthetised with 2% isoflurane (Choongwae Pharma; Seoul, Korea) in 2:1 N_2_O:O_2_ in an anaesthesia chamber. The sciatic nerve was stimulated percutaneously by 1 pulse with a 0.1‐ms duration (PowerLab; ad Instruments, Australia) delivered through a pair of needle electrodes placed at the sciatic notch. Compound muscle action potentials (CMAPs) were recorded with the recording electrode placed subdermally on the muscle belly of the gastrocnemius (GC) and tibialis anterior (TA) muscles. A reference electrode was placed near the Achilles tendon, and a ground electrode was placed at the rat tail. Disposable mono‐polar needle electrodes were used for both stimulation and recording. To reach supra‐maximal stimulation, stimulation was achieved by turning the intensity controller knob (0–10 mA) until the amplitude of the CMAP response did not increase. The stimulation was further increased by 20% to ensure that the CMAP amplitude reached its maximal response.

### 2.9 Immunohistochemistry for Skeletal Muscle Analysis

Dissected muscle tissue was embedded in Tissue Teck O.C.T. compound (Sakura Finetek, Torrance, CA, USA), and all tissue preparation protocols were performed according to previous study [9], and the tissue was stored at −80°C prior to sectioning. The muscles were sectioned at a thickness of 10 μm. For immunohistochemistry, frozen sections were incubated with 1× phosphate‐buffered saline (PBS) for 5 min, washed with 1× PBS, and blocked with 2% normal goat serum in 1× PBS for 1 h. Primary antibodies were diluted in 2% normal goat serum, and the sections were incubated with the antibodies overnight at 4°C. The primary antibodies used are listed in Table S1. The sections were washed in 1× PBS and incubated for 1 h with fluorescent secondary antibodies in 2% normal goat serum. The sections were washed three times for 5 min with 1× PBS, mounted with fluorescence mounting medium (Dako; Cytomation, Carpinteria, CA, USA), and imaged using an EVOS M7000 microscope (Thermo Fisher Scientific; Waltham, MA, USA).

Muscle fibre cross‐sectional area (CSA) was measured by staining with anti‐dystrophin and capturing images at 200× magnification. ImageJ software was used to manually trace each fibre, and two images per individual were analysed. For each individual, 40 fibres were analysed from two images on two slides to determine the mean muscle fibre CSA. Cytochrome C area (CCA) was measured by staining with anti‐cytochrome C and capturing images at 200× magnification using a fluorescence microscope. The total area of fluorescence was measured using Celleste 6 Image Analysis Software (Thermo Fisher Scientific; Waltham, MA, USA). Four images from two slides per individual were analysed, and the average was calculated for each group. Muscle fibre type quantification was performed by staining sections with anti‐MYH7 (Type I, blue), anti‐MYH2 (Type IIa, green), and anti‐MYH4 (Type IIb, red). Images were captured at 100× magnification and analysed using Celleste 6 Image Analysis Software. The total area of each fluorescence signal was measured and divided by the number of fibres stained with each antibody to calculate the average area. Two images from two slides per individual were analysed, and the average was calculated for each group.

### 2.10 Western Blot Analysis

C2C12 myotubes and TA and gastrocnemius (GC) muscles were lysed with cold RIPA buffer (10 mM Tris–HCl, pH 8.0; 1 mM EDTA; 0.5 mM EGTA; 1% Triton X‐100; 0.1% SDC; 0.1% SDS; and 140 mM NaCl) supplemented with 1% protease inhibitor cocktail and 1% phosphatase inhibitor cocktail (Sigma–Aldrich). Whole‐cell lysates were centrifuged at 15 000× *g* for 15 min, and the supernatants were transferred to new tubes. The protein concentration of each sample was quantified using a Bradford protein assay kit (Bio‐Rad; Hercules, CA, USA). Equal amounts of protein (70 μg) were separated by SDS–PAGE and transferred to polyvinylidene difluoride (PVDF) membranes (GE Healthcare; Marlborough, MA, USA), and the membranes were blocked with 5% skim milk or 5% bovine serum albumin (BSA) in 0.1% Tween‐20 Tris‐buffered saline (TBST) buffer for 1 h at room temperature, followed by incubation with specific primary antibodies overnight at 4°C. After washing with TBST three times, a horseradish peroxidase (HRP)‐conjugated mouse IgG or rabbit IgG secondary antibody was added, and the membrane was incubated for 1 h at room temperature. Then, the cells were exposed to the reagents of an enhanced chemiluminescence (ECL) kit (GE‐Health Care). Specific protein bands were visualized using an iBright 1500 (Invitrogen; Waltham, MA, USA). The intensity of individual bands in the western blots was quantified using ImageJ (ImageJ; Bethesda, MD, USA).

### 2.11 Quantitative Real‐Time Polymerase Chain Reaction

Total RNA was isolated from C2C12 cells using an Easy‐spin Total RNA Extraction Kit (iNtRON, Seoul, Korea). Subsequently, cDNA was synthesized from the extracted RNA using the ReverTra Ace quantitative real‐time polymerase chain reaction (qPCR‐RT) kit (Toyobo, Osaka, Japan). Quantitative real‐time PCR (qRT–PCR) was carried out on a StepOne system (Applied Biosystems) with the iTaq Universal SYBR Green Supermix (Bio‐Rad, CA, USA). The specific primers used in the experiment can be found in Table S2. During qRT–PCR, a dissociation curve was generated within the temperature range of 65°C to 95°C. The cycling conditions consisted of an initial cycle at 95°C for 30 s for polymerase activation and DNA denaturation, followed by 40 amplification cycles of 15 s at 95°C and 1 min at 55°C–60°C. The threshold cycle was recorded, and the relative expression of each target gene was analysed.

### 2.12 RNA‐Sequencing Analysis

RNA was purified using an RNeasy kit (Qiagen; Hilden, Germany). To construct the library for sequencing, approximately 200 ng of total RNA was used for library construction using the MGIEasy RNA Directional Library Prep Set V2.0 (MGI; Shenzhen, China). Next, paired‐end sequencing was performed using the DNBSEQ‐T7 sequencing instrument according to the manufacturer's instructions, yielding 150 bp paired‐end reads. The sequences were aligned to the *Rattus norvegicus* genome assembly Rnor_6.0 using STAR version 2.7.3a. RSEM version 1.3.1 was used in combination with the STAR program. STAR and RSEM were set as default parameters.

### 2.13 DEG Analysis and Functional Classification

Differentially expressed genes and enrichment were analysed with DESeq2 for DEG identification from RNA‐seq data that were normalized to expected counts at a log2FC ≥ 1 or ≤ − 1 and q‐value < 0.01. The overall expression pattern was visualized through scatter plots, MA plots, violin plots, heatmaps and principal component analysis plots using the ggplot2 R package. Heatmap clustering analysis of DEGs was performed based on log2 FPKM values using the hclust2 package (v3.6.2; available at https://github.com/SegataLab/hclust2), employing Pearson's distance for clustering distance calculation and the complete linkage method for hierarchical clustering. To facilitate biological interpretation, the Reactome pathway database, which can be used to interpret biological pathways, was used to identify the functional role of genes that show differences in gene expression depending on treatment status. Meta‐analysis of multiple gene lists was performed using the Metascape (https://Metascape.org/) tool to predict various biological pathways that were significantly related to the gene set identified in this study (with an enrichment factor of 1.5 and a *p* value threshold of 0.01 for filtering purposes).

### 2.14 Statistical Analysis

All numeric data are reported as means ± standard deviations, and SPSS Statistics 26 (International Business Machines Corp.; Armonk, NY) was used for all analyses. The Shapiro–Wilk test was performed to confirm the normal distribution of all quantitative histological and functional data from each group, and according to the results, parametric or nonparametric tests were chosen. We evaluated significant differences among the control and experimental groups using one‐way analysis of variance (ANOVA) and the Games–Howell post hoc test. Repeated measures two‐way ANOVA (time and group) and Bonferroni correction were used to compare the changes in the pharyngeal pumping rate of *C. elegans* and body weight and grip strength of the rats in the control and experimental groups, and Bonferroni post hoc correction was subsequently used to analyse the data from each time point. The effects were considered significant at *p* < 0.05.

## 3 Results

### 3.1 Lonafarnib Improves Recovery From Muscle Atrophy in Dexamethasone‐Induced Muscle Atrophy Models in Vitro

In this study, we established a dexamethasone‐induced muscle atrophy model using C2C12 myoblasts, which exhibited a significantly lower myotube area, fusion index and myotube diameter compared to those of control cells (Figure 1a–d; control (CTL) vs. dexamethasone‐induced muscle atrophy model (DEX) when stained with MYH (green) and DAPI (blue). These dexamethasone‐induced C2C12 myoblasts were treated with different concentrations of lonafarnib (0.5, 1, 2 or 4 μM). Notably, 2 μM lonafarnib effectively restored the myotube area and fusion index to levels comparable with those of the control group (Figure 1a–c). In addition, an increase in myotube diameter was observed after treatment with 1 or 2 μM lonafarnib (Figure 1a,d). Further molecular analysis using real‐time PCR revealed that the expression levels of the atrophy‐related genes MuRF1 and MAFbx were increased in the dexamethasone‐induced models. However, these levels returned to control levels upon treatment with 2 μM lonafarnib (Figure 1e,f).

### 3.2 Lonafarnib Improves Motor Function and Muscle Mass in Animal Models of Muscle Atrophy

**FIGURE 2:**
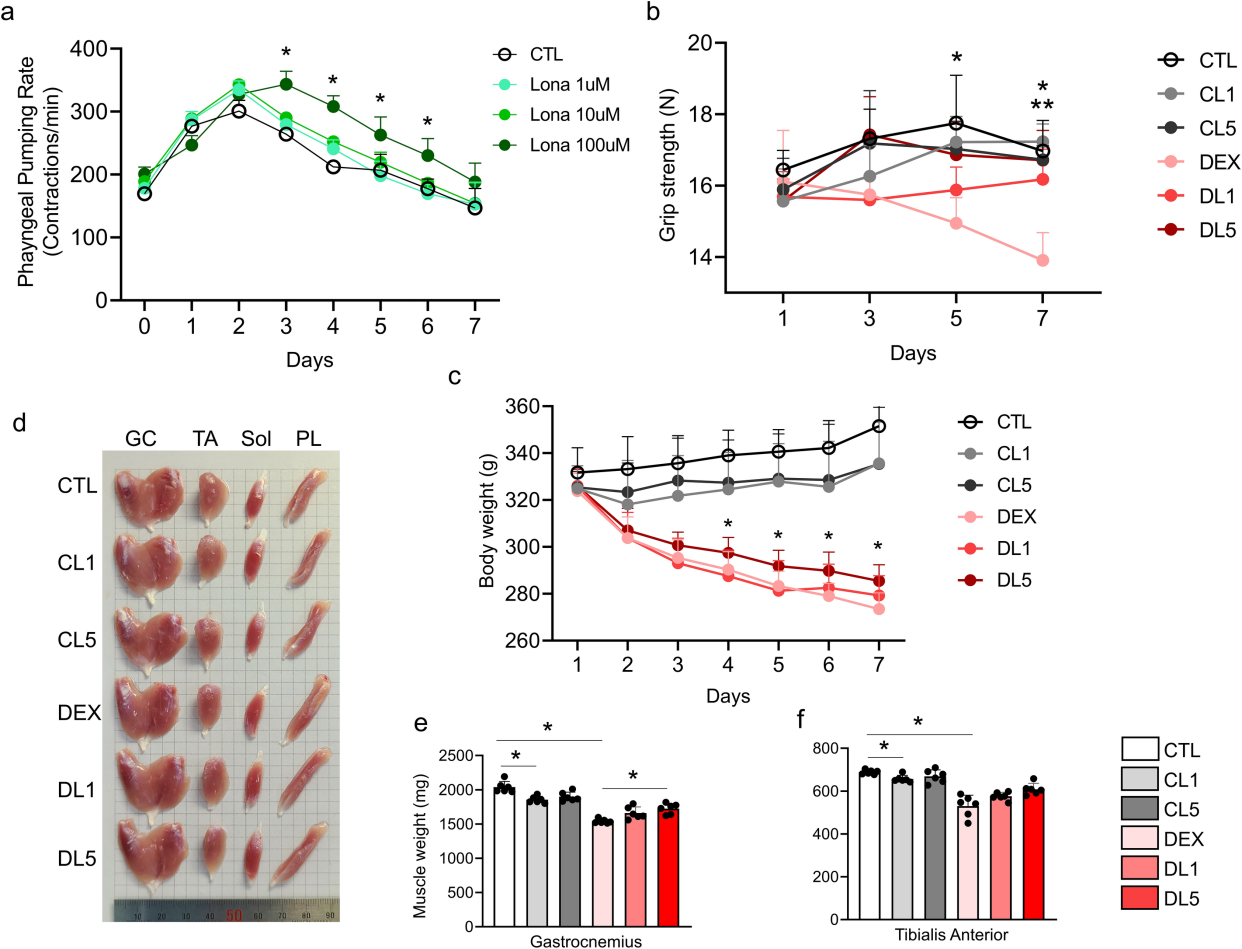
Effects of lonafarnib on motor function and muscle weight in animal models of muscle atrophy. (a) Pharyngeal pumping velocity in *C. elegans* : Comparison between the control (CTL) group and the groups treated with 1, 10 or 100 μM lonafarnib (*n* = 3 per group). (b) Grip strength results:Comparison between control (CTL) and dexamethasone (DEX)‐induced muscle atrophy model rats treated with or without 1 or 5 μM lonafarnib (CL1, CL5, DL1, or DL5) (*n* = 6 per group). (c) Changes in body weight: Analysis of control and dexamethasone‐induced muscle atrophy model rats treated with or without 1 or 5 μM lonafarnib. (d) Representative images of muscle tissue from each group (*n* = 6 per group). Weights of specific hindlimb muscles—the gastrocnemius (e), and tibialis anterior (f)—across groups (*n* = 6 per group). *p < 0.05 between the control and group treated with 100 μM lonafarnib (a), and between DEX group and group treated with 5 μM lonafarnib (DL5) (b, c), and **p < 0.05 between DEX group and group treated with 1 μM lonafarnib (DL1) (b) by two‐way ANOVA followed by the Bonferroni post hoc test.. *p < 0.05 by one‐way ANOVA with the Games–Howell post hoc test (e, f). CTL = control, CL1 = control that received 1 μM lonafarnib, CL5 = control that received 5 μM lonafarnib, DEX = dexamethasone‐induced muscle atrophy model, DL1 = dexamethasone‐induced muscle atrophy model that received 1 μM lonafarnib, DL5 = dexamethasone‐induced muscle atrophy model that received 5 μM lonafarnib.

In our study of muscle atrophy using both *C. elegans* and rat models, we observed significant therapeutic effects of lonafarnib. In *C. elegans* , a model of natural aging, the administration of 100 μM lonafarnib significantly increased the pharyngeal pumping rate, an indicator of improved neuromuscular function, from day 3 to day 7, as shown in Figure 2a. This result highlights the potential of lonafarnib to improve neuromuscular function in aging individuals. In parallel, rats with dexamethasone‐induced muscle atrophy treated with 5 μM lonafarnib exhibited a marked improvement in grip strength on day 5 and 7, and those treated with 1 μM lonafarnib on day 7 (Figure 2b) and a significant recovery of body weight beginning on day 4 (Figure 2c). In addition, lonafarnib effectively counteracted dexamethasone‐induced muscle weight loss, specifically increasing the weight of the gastrocnemius muscle at 5 μM (Figure 2e) and the weight of the plantaris muscle at both 1 and 5 μM (Figure S1f). A significant increase in the ratio of gastrocnemius muscle weight to total body weight was observed in the groups treated with 5 μM lonafarnib (Figure S1a), while other muscles, such as the tibialis anterior, soleus, and plantaris muscles, did not show similar changes in relative weight (Figure S1b–d). Together, the findings from Figure 2a–c,e, and Figures S1f and S1a–d highlight the efficacy of lonafarnib in not only increasing muscle mass but also improving motor function in models of aging, positioning it as a promising therapeutic agent for muscle atrophy.

### 3.3 Lonafarnib Improves Electrophysiological Functions in Animal Models of Muscle Atrophy

**FIGURE 3:**
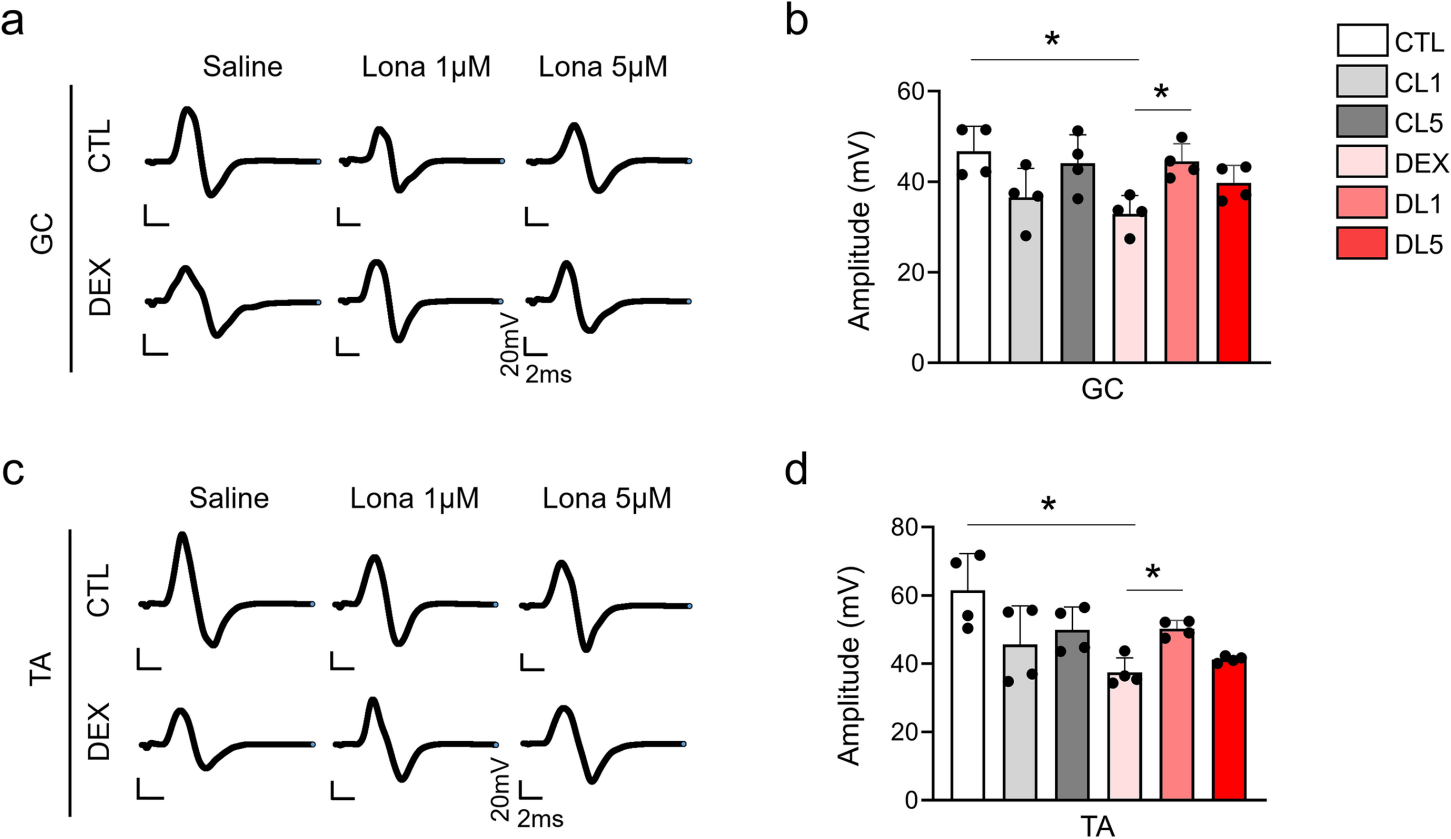
Effect of lonafarnib on electrophysiological function in muscle atrophy models. Representative images showing the compound muscle action potential (CMAP) in the gastrocnemius (GC) (a) and tibialis anterior (TA) (c) muscles in different groups: control (CTL), dexamethasone‐induced muscle atrophy (DEX) models, and models treated with 1 or 5 μM lonafarnib (CL1, CL5, DL1 and DL5, respectively) (*n* = 4 per group). Quantitative analysis of onset latency in the gastrocnemius (b) and tibialis anterior (d) muscles. *p < 0.05 by one‐way ANOVA with the Games–Howell post hoc test. GC = gastrocnemius, TA = tibialis anterior.

As we established the effects of lonafarnib on motor functions, we further investigated the electrophysiological aspects of its effects on muscle functions. The results revealed significant differences in compound muscle action potential (CMAP) amplitudes (Figure 3). In dexamethasone‐induced muscle atrophy (DEX) models, both the gastrocnemius (Figure 3a,b) and tibialis anterior (Figure 3c,d) muscles showed significantly lower CMAP amplitudes than did those in the control group (CTL). Importantly, those lower CMAP amplitudes were rescued by lonafarnib treatment, particularly at 1 μM, with the higher (5 μM) concentration group also showing the same trend, without statistical significance. These results demonstrate the potential of lonafarnib to restore electrophysiological functions impaired by muscle atrophy. Other less significant electrophysiological parameters, such as the latency and duration of CMAPs, were not different among the groups (Figure S2).

### 3.4 Lonafarnib Induces Muscle Hypertrophy Through Mitochondrial Biogenesis

**FIGURE 4:**
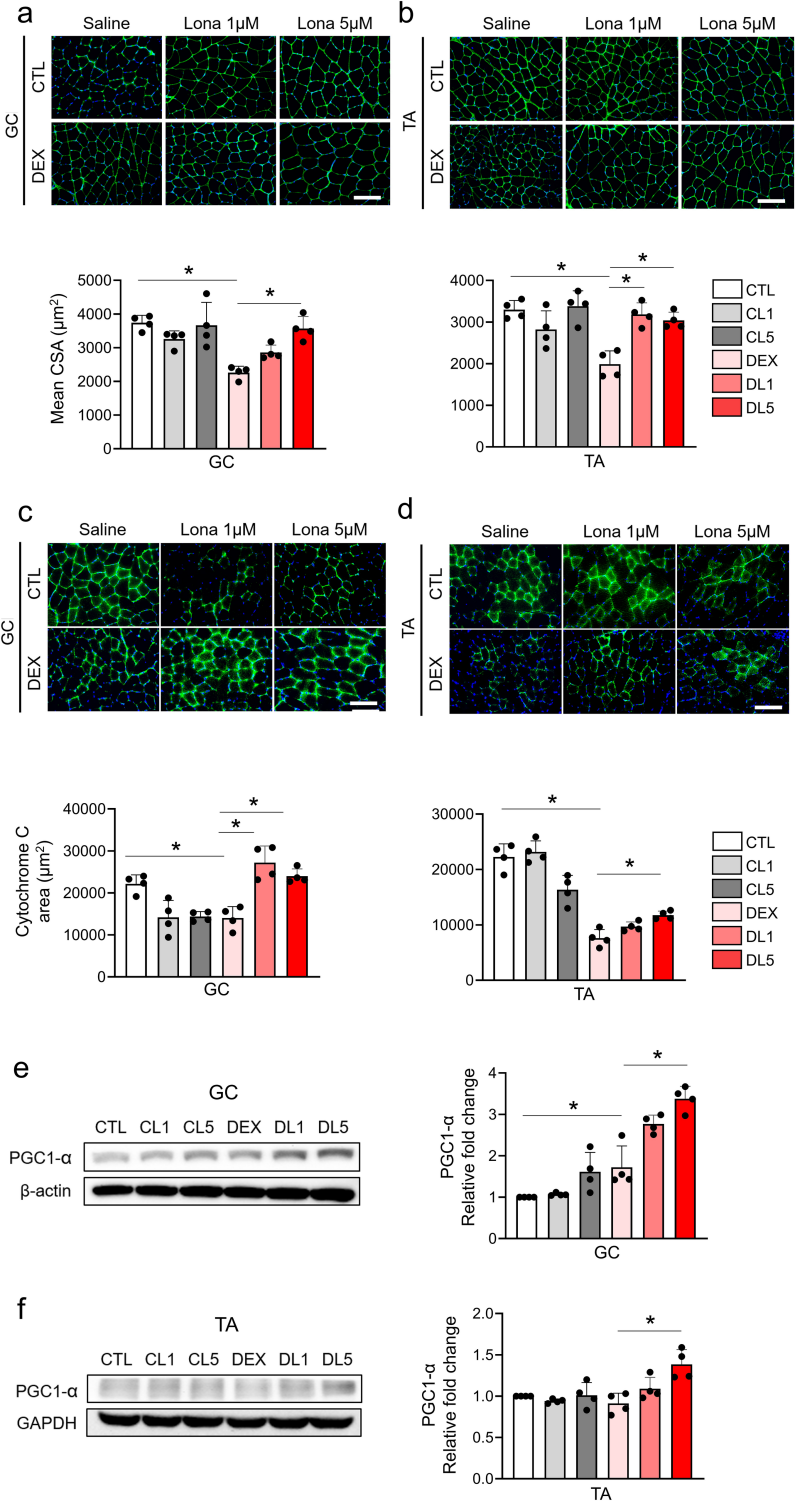
Lonafarnib‐induced histological and molecular changes in muscle atrophy models. Representative images and quantitative analysis of dystrophin‐stained cross‐sectional areas in the gastrocnemius (GC) muscle (a) and tibialis anterior (TA) muscle (b). Representative images and quantitative analysis of cytochrome C‐stained areas in the GC muscle (c) and TA muscle (d) in different groups. Western blot images and relative expression of PGC1‐α in the GC muscle (e) and TA muscle (f) in different groups (*n* = 4 per group). *p < 0.05 by one‐way ANOVA with the Games–Howell post hoc test.

Based on the motor and electrophysiological improvements induced by lonafarnib, we further tested its effect on muscle hypertrophy. The cross‐sectional area (CSA) of the gastrocnemius and tibialis anterior muscles was significantly lower in the muscle atrophy model group than in the control group, as estimated by immunohistochemical staining for dystrophin (Figure 4a,b). Treatment with lonafarnib significantly restored the average CSA of individual myofibres in both the gastrocnemius muscle (Figure 4a) and the tibialis anterior muscle (Figure 4b). As muscle hypertrophy is expected to involve mitochondrial biogenesis, we also measured mitochondrial content in both muscles by cytochrome C staining. The cytochrome C area (CCA) decreased in the muscle atrophy model, suggesting mitochondrial dysfunction. Consistent with the role of CSA in muscle hypertrophy, lonafarnib generally increased the CCA in both the gastrocnemius (Figure 4c) and tibialis anterior (Figure 4d) muscles, suggesting that lonafarnib has beneficial effects on mitochondrial biogenesis. This finding was further corroborated by the increased expression of PGC1‐α, a key regulator of mitochondrial biogenesis, in both muscles after lonafarnib treatment (Figure 4e,f). Therefore, these data show that lonafarnib induces muscle fibre enlargement by enhancing mitochondrial biogenesis.

### 3.5 The Effect of Lonafarnib Is Independent of Muscle Fibre Type

Following the observation of significant functional and histological effects, we extended our investigation to assess whether there are differential effects according to muscle type. With the involvement of mitochondrial biogenesis in the effect of lonafarnib, it is expected that muscle fibres are differentially affected by lonafarnib according to their mitochondrial content. To test this hypothesis, we first characterized the fibre content in GC and TA muscles by immunohistochemistry, which revealed that the GC muscle is rich in all type I, type IIa, type IIb and type × fibres, whereas the TA muscle is predominantly type IIa, type IIb and type × fibres (Figure S5a,b in GC and Figure S5g,h in TA).

The effect of lonafarnib on the CSA of each fibre type was measured, and there was a consistent increase in the CSA regardless of the fibre type (types I, IIa and IIb of GC in Figure S5c–e, and types IIa and IIb of TA in Figure S5i,j, respectively) in both GC and TA muscles in muscle atrophy models. As type I fibres have greater mitochondrial content, these results suggest that mechanisms other than mitochondrial biogenesis are involved in the effect of lonafarnib on muscle hypertrophy.

### 3.6 Lonafarnib Directly or Indirectly Modulates Various Genes and Pathways in Muscle Atrophy Models

**FIGURE 5:**
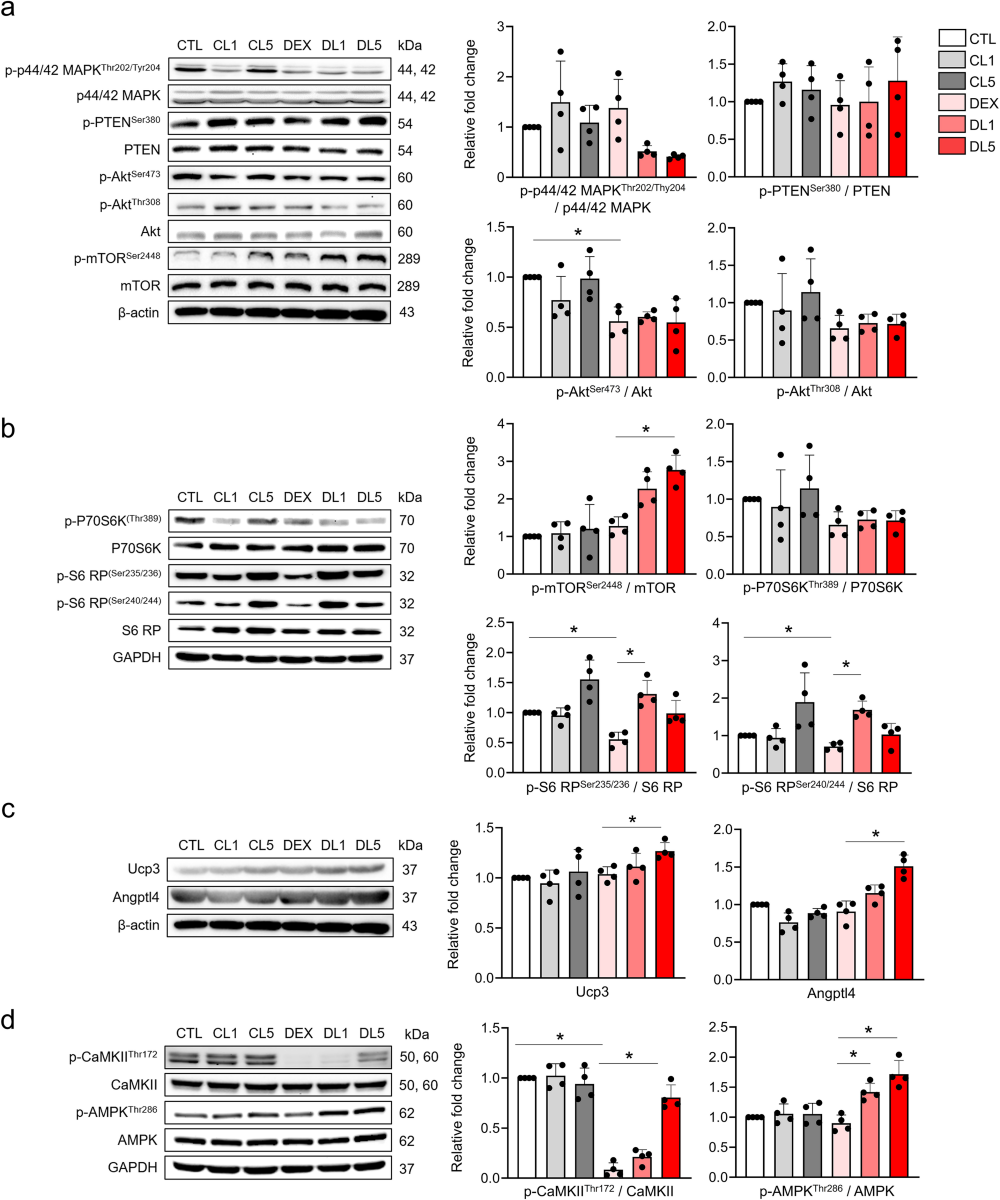
Transcriptional changes following lonafarnib administration. Summary of DEG analysis and significant aspects of transcriptional transitions in the four comparison groups after lonafarnib administration. The number of (a) up‐ and (b) downregulated genes identified in both tissues in the DEX group (DL1 and DL5). Overlapping areas in the Venn diagram represent genes common to every comparison group. The number of genes in the solid line region represents the number of genes in the gastrocnemius muscles, and the number in the dashed line region represents the number of genes in the tibialis anterior muscle. (c) The common DEGs (15 upregulated DEGs and one downregulated DEG) whose expression significantly changed in response to lonafarnib administration were visualized via heatmap clustering. The legend and colour key show the experimental cases and the number of expression values, respectively. (d) The biological roles of 74 up‐ and 29 downregulated genes that were commonly differentially expressed in at least three groups were identified. Red and blue circles indicate the associations of up‐ and downregulated genes, respectively, with darkness indicating significance. (e) A violin plot showing the distribution of the expression intensity of genes involved in six biological mechanisms essential for muscle homeostasis. Violin plots combine box plots and kernel density traces to describe the distribution pattern of a data vector. All expression differences were compared to those of the control to determine whether each group exhibited a drastic change in the corresponding biological mechanism. One to four asterisks (*–****) indicate *p* values less than 0.05, 0.01, 0.001, and 0.0001, respectively.

**FIGURE 6:**
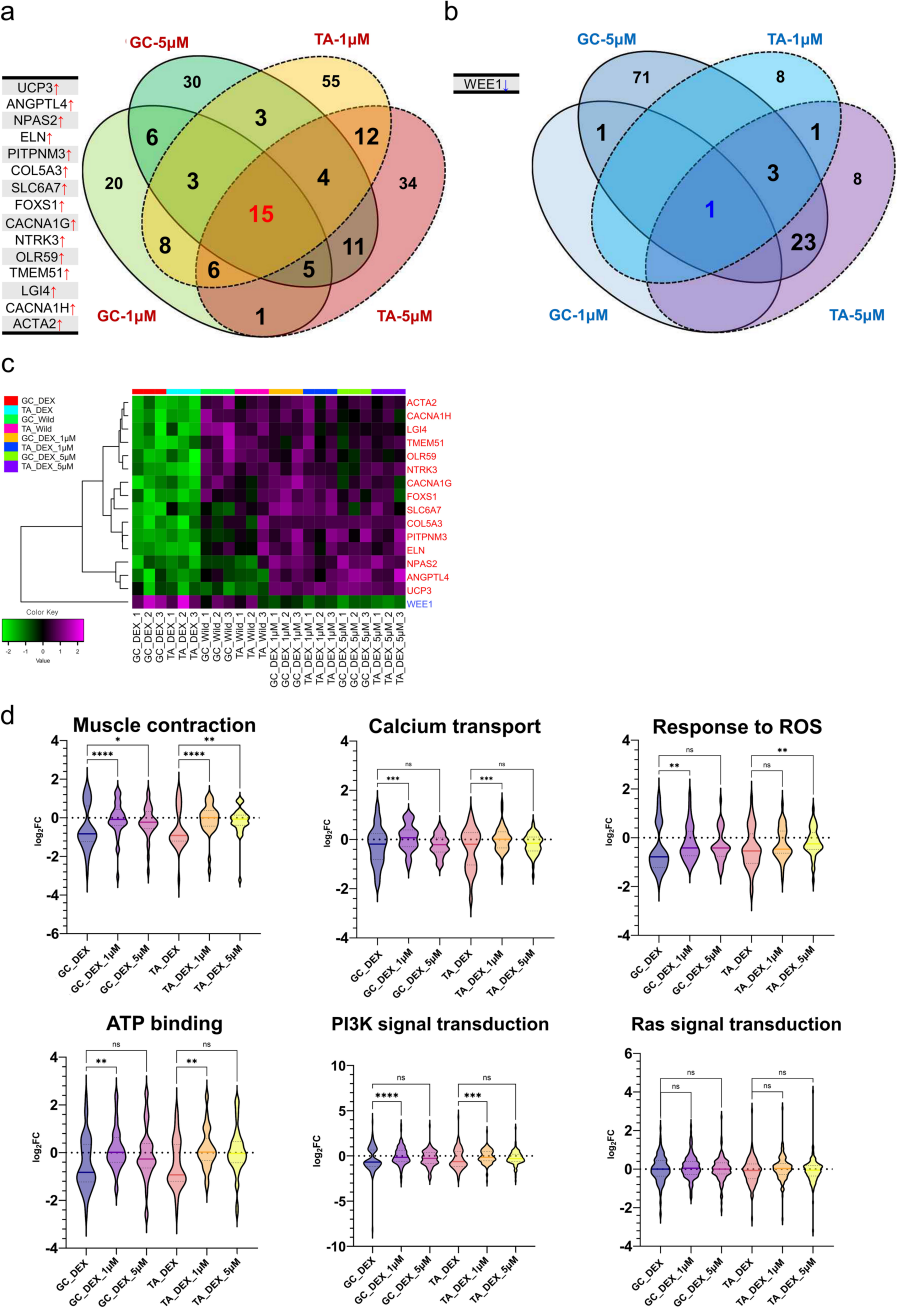
Western blot analysis of key genes in muscle atrophy models after treatment with lonafarnib. (a) Representative images of western blots (left) and quantitative analysis of the phosphorylated p44/42^Thr202/Thy204^(p‐p44/42^Thr202/Thy204^)‐to‐p44/42 MAPK ratio, the phosphorylated PTEN^Ser380^ (p‐PTEN^Ser380^)‐to‐PTEN ratio, the p‐Akt^Ser473^‐to‐Akt ratio, p‐Akt^Thr308^‐to‐Akt ratio and the p‐mTOR^Ser2448^‐to‐mTOR ratio (right). (b) Representative western blot images (left) and quantitative analysis of the phosphorylated P70S6K^Thr389^/P70S6K ratio, the phosphorylated S6 ribosomal protein (RP)^Ser235/236^/S6 RP ratio, and the phosphorylated S6 RP^Ser240/244^/S6 RP ratio (right), (c) representative western blot images (left) and quantitative analysis of Ucp3 and Angptl4 expression (right), and (d) representative western blot images (left) and quantitative analysis of the phosphorylated CaMKII^Thr172^/CaMKII ratio (p‐CaMKII/CaMKII) and phosphorylated AMPK^Thr286^/AMPK ratio (p‐AMPK/AMPK) (right) (*n* = 4 per group). *p < 0.05 by one‐way ANOVA with the Games–Howell post hoc test.

To study the detailed mechanisms of lonafarnib, as suggested above, we performed mRNA sequencing on muscle tissues obtained from control or lonafarnib‐treated (1 or 5 μM) animals. The results, validated by principal component analysis (PCA), revealed transcriptional changes between untreated (DEX) and lonafarnib‐treated muscle atrophy models (Figures S3 and S4, Tables S1 and S2). We then investigated the gene expression changes and looked for genes that were consistently modulated in both the GC and TA muscles at both concentrations of lonafarnib. The significantly altered genes included one downregulated gene (*Wee1*) and 15 upregulated genes, such as Ucp3, Angplt4 and Npas2, which are critical for muscle structure and function (Figure 5a–c). We also performed pathway‐centric analysis using ClueGo, which identified six biological pathways essential for muscle homeostasis (Figure 6d); these included pathways involved in muscle contraction, calcium transport, the ROS response, ATP binding and PI3K signalling, underscoring the comprehensive effect of lonafarnib on muscle maintenance (Figure 5d). Notably, Ras signalling was not affected, which indicates that lonafarnib may not affect Ras‐related transcription (Figure 5d).

### 3.7 Upc3 and Angplt4 Might Be Key Genes Responsible for the Effects of Lonafarnib

To validate the findings from our RNA‐seq data, we performed extensive Western blot analysis targeting key proteins identified by differential expression and ClueGo pathway analysis. These proteins include those involved in muscle contraction and PI3K signalling pathways (p44/42 MAPK phosphorylated at Thr202/Thy204, PTEN phosphorylated at Ser380, Akt phosphorylated at Ser473 and Thr308, mTOR phosphorylated at Ser2448, P70S6K phosphorylated at Thr389, S6 ribosomal protein [S6 RP] phosphorylated at Ser235/236 and Ser240/244), calcium transport pathways (CaMKII phosphorylated at Thr172 and AMPK phosphorylated at Thr286), and proteins from our differential gene expression analysis (UCP3 and ANGPTL4). In gastrocnemius muscle, treatment with lonafarnib significantly increased the phosphorylation levels of mTOR at Ser2448 (p‐mTOR^Ser2448^/mTOR) and the phosphorylation of S6 RP at Ser235/236 and Ser240/244 (S6 RP^Ser235/236^/S6 RP and S6 RP^Ser240/244^/S6 RP) in the dexamethasone‐induced muscle atrophy model (Figure 6a,b). These results are consistent with the known roles of mTOR and S6 RP in promoting muscle hypertrophy. However, lonafarnib treatment did not significantly alter the phosphorylation level of Akt at Ser473 (p‐Akt^Ser473^/Akt), which decreased in the muscle atrophy model (Figure 6a,b). In addition, lonafarnib significantly increased the phosphorylation of CaMKII at Thr172 (p‐CaMKII^Thr172^/CaMKII) and AMPK at Thr286 (p‐AMPK^Thr286^/AMPK) in the lonafarnib‐treated muscle atrophy group (Figure 6d), suggesting a potential improvement in calcium transport signalling pathways. Similar results were observed in the tibialis anterior (TA) muscle, except for the phosphorylation levels of mTOR at Ser2448 and CaMKII at Thr172, which did not show similar changes (Figure S7). These results confirm our RNA sequencing results and highlight the efficacy of lonafarnib in modulating known metabolic and signalling pathways in muscle cells. In addition, lonafarnib treatment (5 μM) significantly upregulated UCP3 and ANGPTL4 protein levels in the muscle atrophy group (Figure 6c), consistent with the changes observed in the gene expression profiles. Western blot analysis of TA muscle confirmed the absence of changes in p44/42 MAPK levels and a consistent decrease in the relative levels of p‐Akt/Akt, similar to the findings in gastrocnemius muscle (Figure S7). H‐Ras levels remained unchanged after lonafarnib application, consistent with mRNA sequencing analysis (Figures S6 and S7e).

Collectively, these results suggest that lonafarnib promotes muscle hypertrophy by enhancing the expression and activation of key signalling proteins involved in protein synthesis pathways, such as mTOR^Ser2448^, S6 RP^Ser235/236^ and S6 RP^Ser240/244^. While p44/42 MAPK levels were not affected by lonafarnib, its role in muscle signalling networks cannot be completely ruled out, necessitating further investigation into the broader regulatory mechanisms.

**FIGURE 7:**
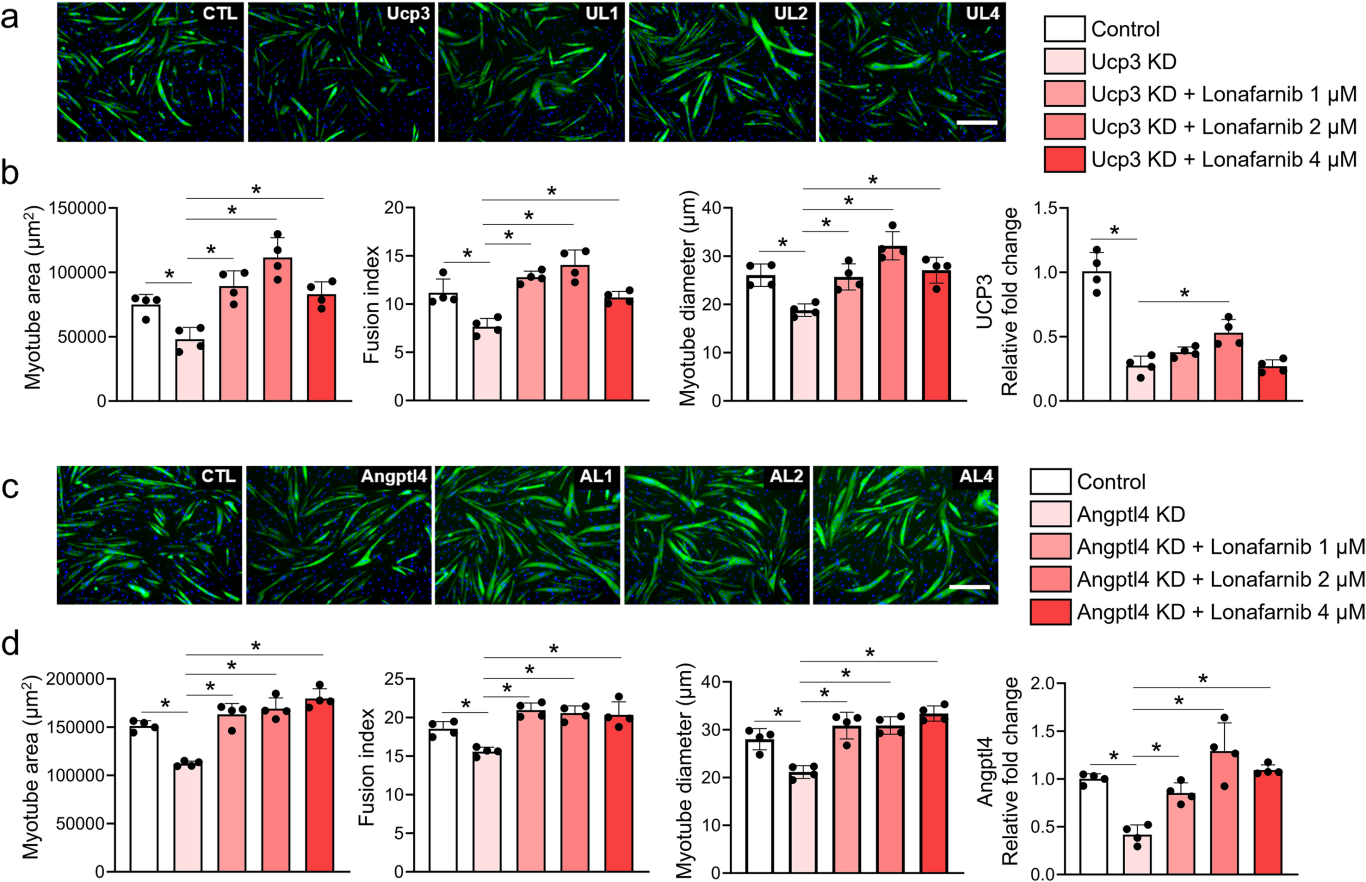
Effect of lonafarnib in the Ucp3 and Angptl4 knockdown models. Representative image and the results of the quantitative analyses of myotube area, fusion index, myotube diameter, and relative fold changes (RT–PCR) in Ucp3 (a and b, respectively) and Angptl4 (c and d, respectively) knockdown models (*n* = 4 per group). *p < 0.05 by one‐way ANOVA with the Games–Howell post hoc test.

As little is known about the roles of these proteins in muscle functions, we further assessed the involvement of these proteins in the muscle atrophy model phenotype. The knockdown of either Ucp3 or Angptl4 significantly reduced the myotube area, fusion index, and myotube diameter, suggesting their roles in muscle phenotypes (Figure 7). Importantly, treatment with lonafarnib effectively reversed the reductions in myotube size caused by siRNA‐mediated knockdown of UCP3 (Figure 7a,b) and ANGPTL4 (Figure 7c,d). This finding indicates that while ANGPTL4 and UCP3 are important mediators of lonafarnib's effects, other signalling pathways, including those involving mTOR and S6 ribosomal protein, may also contribute to its muscle‐preserving actions. The partial rescue of myotube size in the knockdown models suggests that lonafarnib exerts its protective effects through multiple mechanisms, enhancing its potential as a therapeutic agent against muscle atrophy.

## 4 Discussion

Mitochondrial biogenesis is a complex process that is critical for muscle health and is regulated by a network of signalling pathways and transcription factors. One of the key regulators is PGC1‐α, the expression of which, in our study, was significantly upregulated in the gastrocnemius muscle following lonafarnib treatment [10], suggesting that lonafarnib may enhance mitochondrial biogenesis, potentially improving muscle function in dexamethasone‐induced muscle atrophy. In addition to PGC1‐α, lonafarnib treatment increased the phosphorylation of mTOR at Ser2448 and S6 ribosomal protein at Ser235/236 and Ser240/244, suggesting enhanced anabolic signalling, which is crucial for muscle hypertrophy. Furthermore, we observed an increase in Angptl4 and Ucp3 levels following lonafarnib treatment. Angptl4 has been implicated in lipid metabolism and mitochondrial function [11], while Ucp3 is known to be involved in the regulation of mitochondrial membrane potential and protection against oxidative stress [12, 13]. These findings suggest that lonafarnib may exert multiple effects on mitochondrial function beyond the upregulation of PGC1‐α expression.

Interestingly, our study also revealed an increase in the p‐AMPK/AMPK ratio following lonafarnib treatment [14]. AMPK is a known activator of PGC1‐α, plays a critical role in cellular energy homeostasis and is activated by Angplt4 [15]. The lonafarnib‐induced upregulation of PGC1‐α, Angptl4, Ucp3, and p‐AMPK suggests a coordinated effort to improve mitochondrial function and muscle health. Furthermore, the lonafarnib‐induced increase in PGC1‐α could counteract the degenerative changes associated with muscle atrophy, such as mitochondrial dysfunction, thereby improving muscle function and mass [16].

Another mechanism may contribute to the muscle‐protective effects of lonafarnib. Ca2+ plays a key role in skeletal muscle function, particularly in the contraction‐relaxation cycle [17]. Our molecular analyses, including RNA‐seq, revealed that lonafarnib enriched Ca^2+^‐related factors in both the gastrocnemius and tibialis anterior muscles. This finding is consistent with previous studies indicating that Ca2+ activation can counteract muscle atrophy [18], that Ca2+ inhibition can decrease muscle strength [19] and that lonafarnib activates the CaMKII signalling pathway in the hippocampus and induces functional recovery [20]. AMPK lies downstream of CaMKII in muscle [21], and CaMKII plays an important role in activating mitochondrial biogenesis [22].

In addition to affecting AMPK, UCP3, and ANGPTL4, AMPK is a regulator of UCP3 [23], and UCP3 is not only activated by AMPK but also involved in mitochondrial Ca2+ uptake [24]. Angptl4 is affected by exercise‐induced AMPK activation and plays a role in lipid metabolism and energy homeostasis [23]. Conversely, there is a bidirectional relationship in which the activation of AMPK leads to a decrease in Angptl4 expression [25]. Both proteins are sensitive to changes in intracellular Ca2+ levels [26], suggesting a complex interplay between AMPK, mitochondrial function and calcium signalling [15]. This intricate relationship is further supported by our observation of a lonafarnib‐induced increase in the p‐CaMKII/CaMKII ratio in the gastrocnemius muscle. CaMKII activation has been shown to activate calcineurin, a phosphatase that dephosphorylates and activates transcription factors critical for muscle health [27, 28]. Taken together, these findings suggest that lonafarnib may protect against muscle atrophy through a dual mechanism involving both mitochondrial biogenesis and Ca2+ signalling. This multifaceted strategic mechanism may contribute to muscle homeostasis and hypertrophy, as evidenced by the increased expression of Ucp3 and Angptl4, possibly mediated by AMPK activation.

Farnesyltransferase inhibitors (FTIs), such as lonafarnib, are known primarily for their inhibitory effects on Ras signalling. In cellular systems, Ras signalling has been implicated in the regulation of Akt activity, which in turn can influence muscle physiology. Specifically, Akt activation can promote muscle growth via the mTOR pathway and inhibit muscle atrophy via the FoxO pathway [29]. However, the role of the Ras/Raf/MEK/ERK pathway in skeletal muscle is less clear [30]. Notably, Akt/mTOR signalling is generally activated downstream of the IGF‐I/IGFR/IRS‐1 pathway, which has been shown to be essential for muscle hypertrophy [31]. In our dexamethasone‐induced muscle atrophy models, we did not observe changes in Ras signalling or p44/42 MAPK levels, suggesting that Ras may not be a primary mediator of skeletal muscle hypertrophy or atrophy in this context. Instead, we observed significant effects downstream within the PI3K‐Akt–mTOR pathway, particularly through increased phosphorylation of mTOR and S6 ribosomal protein, which are associated with protein synthesis and muscle hypertrophy [32]. Consequently, the beneficial effects of lonafarnib observed in our study are mediated through the enhancement of anabolic signalling pathways rather than Ras inhibition. While the role of FTIs in Ras/ERK inhibition is well documented [33], our findings suggest that the effects of lonafarnib on muscle atrophy may involve alternative pathways or mechanisms. Therefore, although Ras inhibition should not be completely ruled out, it may play a secondary or synergistic role in the overall effects of lonafarnib on dexamethasone‐induced muscle atrophy. Recent studies suggest potential protective effects of glucocorticoids against sarcopenia in aging mice [S23], but our findings specifically address dexamethasone‐induced muscle atrophy, highlighting the beneficial effects of lonafarnib through the enhancement of mitochondrial function and Ca2+ signalling pathways.

**FIGURE 8:**
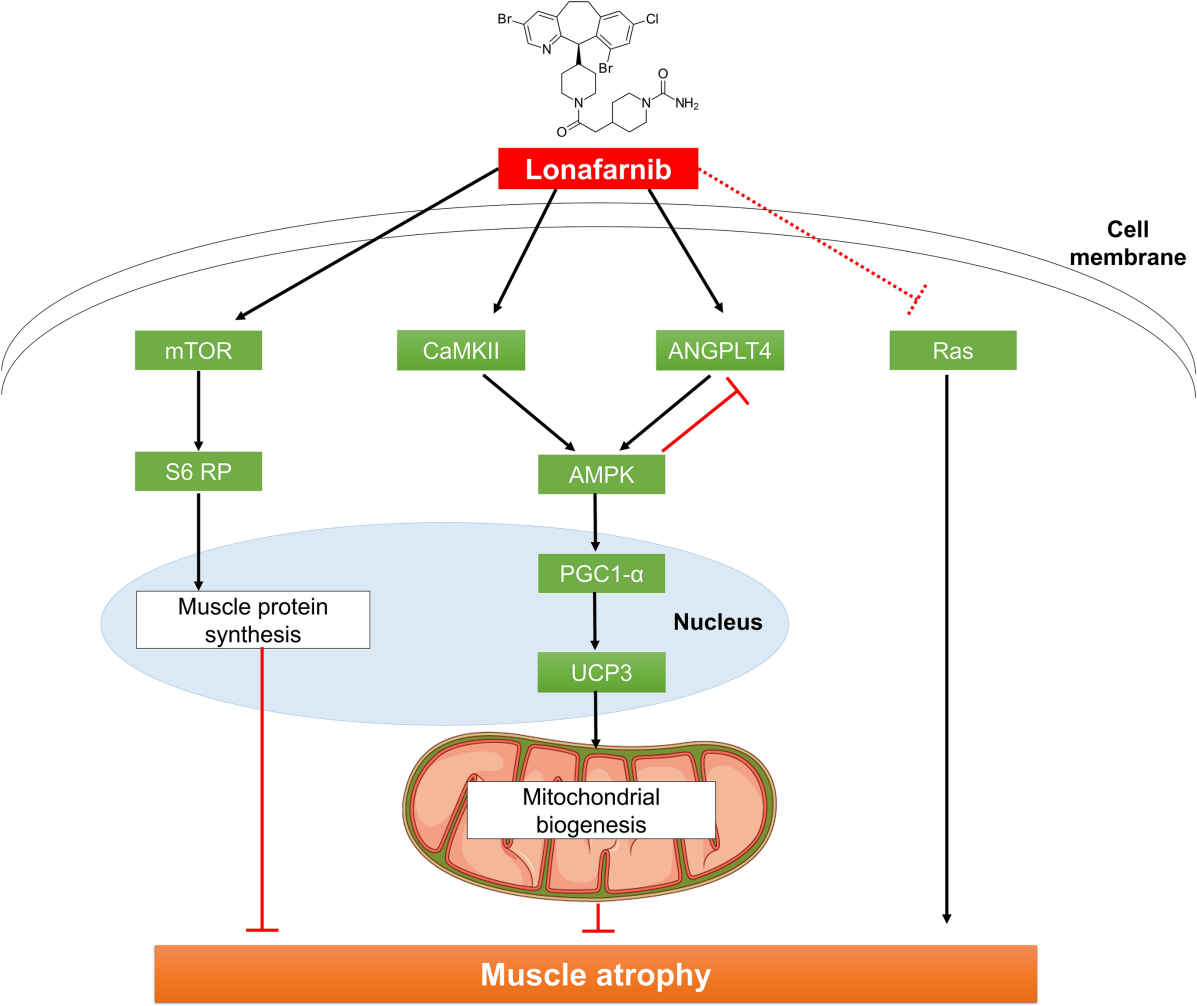
Proposed mechanisms of action of lonafarnib in muscle atrophy models.

Based on the above discussion, we propose a possible mechanism by which lonafarnib exerts beneficial effects on dexamethasone‐induced muscle atrophy (Figure 8). Lonafarnib appears to improve mitochondrial biogenesis through the upregulation of key regulators such as PGC1‐α, Angptl4, CaMKII, and Ucp3, and promotes muscle hypertrophy through the enhancement of mTOR signalling and S6 ribosomal protein activation.

In the present study, the expression levels of Mib3, Actn3 and PGC1‐α significantly increased in the gastrocnemius muscle, which is predominantly composed of type I fibres, after lonafarnib treatment. Specifically, Mib3 levels were elevated, suggesting a potential role in muscle fibre maintenance and regeneration [34]. Concurrently, Actn3 levels were also increased, a finding that differs from conventional understanding, as Actn3 is generally associated with fast‐twitch fibres [35]. In addition, PGC1‐α, a master regulator of mitochondrial biogenesis, was significantly increased in the gastrocnemius muscle, suggesting improved metabolic efficiency and resistance to muscle atrophy [36]. In contrast, the tibialis anterior muscle, which has a different fibre composition, showed a less pronounced response to lonafarnib treatment. The lack of significant changes in key markers such as PGC1‐α in the tibialis anterior muscle suggests a tissue‐specific effect of lonafarnib, an observation consistent with its documented effects on hepatic and neuronal tissues [37]. This raises the possibility that the effects of the drug may be modulated by inherent characteristics of the muscle tissue, such as fibre type composition [38]. While this study primarily assessed differences in muscle recovery, future research could also consider a non‐inferiority or equivalence framework to further validate the therapeutic potential of lonafarnib [S24].

One of the limitations of this study lies in the use of dexamethasone‐induced models, both in vitro and in vivo, to simulate sarcopenia. Although these models are valuable for studying muscle atrophy, they may not fully recapitulate the complexities of natural aging or the multifactorial aetiology of sarcopenia, which includes cellular senescence, proteostasis imbalance, oxidative stress, and inflammation, as shown in different sarcopenia models [39]. To address this limitation, we employed *C. elegans* as an additional model system. *C. elegans* is a well‐established model for aging research, offering a more natural context for studying age‐related muscle decline [40]. We conducted a pharyngeal pumping assay using varying concentrations of lonafarnib to assess its functional effects. This multimodel approach enriches the translational relevance of our findings, although further studies in more clinically relevant models are warranted. Another limitation is the comparison between different muscle types, as we collected hindlimb muscles for molecular and histological analysis while performing functional assessments on forelimb muscles. Given the distinct fibre composition and functional roles of these muscle groups, our findings should be interpreted with caution. Although similar approaches have been employed in previous studies [S25, S26], the inherent differences between hindlimb and forelimb muscles may influence the outcomes, and this discrepancy underscores the need for careful consideration in future research.

Additionally, while we observed changes in specific signalling pathways, the direct causality between these changes and improvements in muscle function remains to be established. Future research should focus on validating our findings in different animal models and human clinical trials. Investigating the long‐term effects of lonafarnib and its impact on other signalling pathways will also be crucial for understanding its therapeutic potential in muscle atrophy.

## 5 Conclusion

Lonafarnib effectively counteracted dexamethasone‐induced muscle atrophy both in vitro and in vivo, demonstrating its potential as a promising therapeutic candidate for muscle atrophy. We identified several signalling pathways, notably mitochondrial biogenesis and calcium signalling, that warrant further investigation. Although our study has limitations, it lays the groundwork for future research aimed at developing targeted therapies for sarcopenia.

## Conflicts of Interest

The authors declare no conflicts of interest.

## Supporting information


**Table S1.** Antibodies for the immunoassays.
**Table S2.** Primers for the qRT–PCR analysis.
**Table S3.** Summary of the sequencing statistics.
**Table S4.** Summary of the biological processes affected by lonafarnib application.
**Figure S1.** Analysis of the muscle‐to‐body weight ratio shows the ratio of muscle weight to total body weight in various muscles: gastrocnemius (a), tibialis anterior (b), soleus (c), and plantaris (d) muscles. Weights of specific hindlimb muscles— soleus (e), and plantaris (f)—across groups. *p < 0.05 by one‐way ANOVA with the Games–Howell post hoc test. CTL = control, CL1 = control that received 1 μM lonafarnib, CL5 = control that received 5 μM lonafarnib, DEX = dexamethasone‐induced sarcopenia model, DL1 = dexamethasone‐induced sarcopenia model that received 1 μM lonafarnib, DL5 = dexamethasone‐induced sarcopenia model that received 5 μM lonafarnib.
**Figure S2.** Electrophysiological analysis of compound muscle action potentials. Quantitative results of the onset latency and duration of compound muscle action potentials in the gastrocnemius (a, b) and tibialis anterior (c, d) muscles across different groups: control (CTL), dexamethasone‐induced sarcopenia (DEX) models, and models treated with 1 or 5 μM lonafarnib (CL1, CL5, DL1, and DL5).
**Figure S3.** Bioinformatics analysis of the RNA‐seq data and sample correlations. (a) The boxplot shows the gene expression in each sample after data normalization. The ordinate represents the gene expression value, and the abscissa represents the control, DEX, DL1, and DL5 groups in both GC and TA muscle with independent colours. The distribution of FPKM values for total expressed genes in the samples of each group is also shown. (b) The PCA plot shows how similar and close the transcriptome changes in each sample are based on the global gene expression level. (c) Heatmap clustering analysis of globally expressed genes is shown. The histogram in the colour key at the top shows the expression values.
**Figure S4.** Statistical comparison of DEGs in both tissues between the DEX group and the DEX group. The numbers of up‐ and downregulated genes identified in the four comparison sets are shown: (a) GC‐DL1, (b) GC‐DL5, (c) TA‐DL1, and (d) TA‐DL5. The FPKM volcano plot and MA plot were constructed by pairwise comparison against DEX gene expression. In this statistical analysis, red and blue dots represent the statistically significant up‐ and downregulated DEGs, respectively. The middle line with black dots indicates no difference in the mean expression values between samples. (e) Hierarchical clustering analysis of genes commonly identified in at least three comparison groups in the comparison of DEG and DEX groups for each tissue.
**Figure S5.** Effect of lonafarnib on muscle fibre type and gene expression. Representative images (a) and quantitative analysis of cross‐sectional areas of type I (blue), type IIa (green), type IIb (red), and type X (black) fibres in the gastrocnemius muscle in each group (b) and myotube areas of type I (c), type IIa (d), type IIb (e), and type X (f) fibres in the gastrocnemius muscle in each group. Representative images (g) and quantitative analysis of cross‐sectional areas of type IIa (green) and type IIb (red) fibres in the tibialis anterior muscle (h) and myotube areas of type I (i), type IIa (j), and type IIb (k) fibres in the tibialis anterior muscle in each group. *p < 0.05 compared to the control (CTL) or sarcopenia (DEX) groups by one‐way ANOVA with the Games‐Howell post hoc test.
**Figure S6.** Western blot analysis of key genes in sarcopenia models after treatment with lonafarnib. Representative images of western blots (a) and quantitative analysis of H‐Ras (b). *p < 0.05 by one‐way ANOVA with the Games–Howell post hoc test.
**Figure S7.** Western blot analysis of key genes in sarcopenia models after treatment with lonafarnib. (a) Representative images of western blots (left) and quantitative analysis of the phosphorylated p44/42^Thr202/Thy204^(p‐p44/42^Thr202/Thy204^)‐to‐p44/42 MAPK ratio, the phosphorylated PTEN^Ser380^ (p‐PTEN^Ser380^)‐to‐PTEN ratio, the p‐Akt^Ser473^‐to‐Akt ratio, p‐Akt^Thr308^‐to‐Akt ratio and the p‐mTOR^Ser2448^‐to‐mTOR ratio (right). (b) Representative western blot images (left) and quantitative analysis of the phosphorylated P70S6K^Thr389^/P70S6K ratio, the phosphorylated S6 RP^Ser235/236^/S6 RP ratio, and the phosphorylated S6 RP^Ser240/244^/S6 RP ratio (right), (c) representative western blot images (left) and quantitative analysis of Ucp3 and Angptl4 expression (right), (d) representative western blot images (left) and quantitative analysis of the phosphorylated CaMKII^Thr172^/CaMKII ratio (p‐CaMKII/CaMKII) and phosphorylated AMPK^Thr286^/AMPK ratio (p‐AMPK/AMPK) (right), and (e) representative images of western blots (left) and quantitative analysis of H‐Ras (right). *p < 0.05 by one‐way ANOVA with the Games–Howell post hoc test.

## References

[1] A. J. Cruz‐Jentoft and A. A. Sayer , “Sarcopenia,” Lancet 393 (2019): 2636–2646. 31171417 10.1016/S0140-6736(19)31138-9

[2] Y. Feike , L. Zhijie , and C. Wei , “Advances in Research on Pharmacotherapy of Sarcopenia,” Aging Medicine (Milton) 4 (2021): 221–233. 10.1002/agm2.12168 PMC8444957 34553120

[3] M. Suzuki , L. J. B. Jeng , S. Chefo , et al., “FDA Approval Summary for Lonafarnib (Zokinvy) for the Treatment of Hutchinson‐Gilford Progeria Syndrome and Processing‐Deficient Progeroid Laminopathies,” Genetics in Medicine 25 (2023): 100335. 36507973 10.1016/j.gim.2022.11.003

[4] H. Nakazawa , L. P. Wong , L. Shelton , R. Sadreyev , and M. Kaneki , “Farnesysltransferase Inhibitor Prevents Burn Injury‐Induced Metabolome Changes in Muscle,” Metabolites 12 (2022): 800. 36144205 10.3390/metabo12090800 PMC9506277

[5] R. A. Saxton and D. M. Sabatini , “mTOR Signaling in Growth, Metabolism, and Disease,” Cell 168 (2017): 960–976. 28283069 10.1016/j.cell.2017.02.004 PMC5394987

[6] E. Castellano and J. Downward , “RAS Interaction With PI3K: More Than Just Another Effector Pathway,” Genes & Cancer 2 (2011): 261–274. 21779497 10.1177/1947601911408079 PMC3128635

[7] C. S. E. Hendrikse , P. M. M. Theelen , P. van der Ploeg , et al., “The Potential of RAS/RAF/MEK/ERK (MAPK) Signaling Pathway Inhibitors in Ovarian Cancer: A Systematic Review and Meta‐Analysis,” Gynecologic Oncology 171 (2023): 83–94. 36841040 10.1016/j.ygyno.2023.01.038

[8] S. Brenner , “The Genetics of Caenorhabditis Elegans,” Genetics 77 (1974): 71–94. 4366476 10.1093/genetics/77.1.71 PMC1213120

[9] H. Meng , P. M. Janssen , R. W. Grange , et al., “Tissue Triage and Freezing for Models of Skeletal Muscle Disease,” Journal of Visualized Experiments: JoVE 15 (2014): 51586. 10.3791/51586 PMC4215994 25078247

[10] J. Lin , H. Wu , P. T. Tarr , et al., “Transcriptional Co‐Activator PGC‐1 Alpha Drives the Formation of Slow‐Twitch Muscle Fibres,” Nature 418 (2002): 797–801. 12181572 10.1038/nature00904

[11] Y. Wang , K. S. Lam , J. B. Lam , et al., “Overexpression of Angiopoietin‐Like Protein 4 Alters Mitochondria Activities and Modulates Methionine Metabolic Cycle in the Liver Tissues of db/db Diabetic Mice,” Molecular Endocrinology 21 (2007): 972–986. 17213385 10.1210/me.2006-0249

[12] B. S. Monteiro , L. Freire‐Brito , D. F. Carrageta , P. F. Oliveira , and M. G. Alves , “Mitochondrial Uncoupling Proteins (UCPs) as Key Modulators of ROS Homeostasis: A Crosstalk Between Diabesity and Male Infertility?,” Antioxidants (Basel) 10 (2021): 1746. 34829617 10.3390/antiox10111746 PMC8614977

[13] E. Barreiro , C. Garcia‐Martinez , S. Mas , et al., “UCP3 Overexpression Neutralizes Oxidative Stress Rather Than Nitrosative Stress in Mouse Myotubes,” FEBS Letters 583 (2009): 350–356. 19101552 10.1016/j.febslet.2008.12.023

[14] D. G. Hardie , F. A. Ross , and S. A. Hawley , “AMPK: A Nutrient and Energy Sensor That Maintains Energy Homeostasis,” Nature Reviews Molecular Cell Biology 13 (2012): 251–262. 22436748 10.1038/nrm3311 PMC5726489

[15] H. Chang , O. Kwon , M.‐S. Shin , et al., “Role of Angptl4/Fiaf in Exercise‐Induced Skeletal Muscle AMPK Activation,” Journal of Applied Physiology 125 (2018): 715–722. 29952246 10.1152/japplphysiol.00984.2016

[16] E. Marzetti , R. Calvani , M. Cesari , et al., “Mitochondrial Dysfunction and Sarcopenia of Aging: From Signaling Pathways to Clinical Trials,” International Journal of Biochemistry & Cell Biology 45 (2013): 2288–2301. 23845738 10.1016/j.biocel.2013.06.024 PMC3759621

[17] B. R. MacIntosh , “Role of Calcium Sensitivity Modulation in Skeletal Muscle Performance,” Physiology 18 (2003): 222–225. 10.1152/nips.01456.2003 14614153

[18] N. Ito , U. T. Ruegg , A. Kudo , Y. Miyagoe‐Suzuki , and T. Si , “Activation of Calcium Signaling Through Trpv1 by nNOS and Peroxynitrite as a Key Trigger of Skeletal Muscle Hypertrophy,” Nature Medicine 19 (2013): 101–106. 10.1038/nm.3019 23202294

[19] O. Delbono , J. Xia , S. Treves , et al., “Loss of Skeletal Muscle Strength by Ablation of the Sarcoplasmic Reticulum Protein JP45,” National Academy of Sciences of the United States of America 104 (2007): 20108–20113. 10.1073/pnas.0707389104 PMC2148430 18077436

[20] T. Chen , C. Cai , L. Wang , S. Li , and L. Chen , “Farnesyl Transferase Inhibitor Lonafarnib Enhances α7nAChR Expression Through Inhibiting DNA Methylation of CHRNA7 and Increases α7nAChR Membrane Trafficking,” Frontiers in Pharmacology 11 (2020): 589780. 33447242 10.3389/fphar.2020.589780 PMC7801264

[21] M. A. Raney and L. P. Turcotte , “Evidence for the Involvement of CaMKII and AMPK in Ca‐Dependent Signaling Pathways Regulating FA Uptake and Oxidation in Contracting Rodent Muscle,” Journal of Applied Physiology 104 (2008): 1366–1373. 18309092 10.1152/japplphysiol.01282.2007

[22] E. R. Chin , “The Role of Calcium and Calcium/Calmodulin‐Dependent Kinases in Skeletal Muscle Plasticity and Mitochondrial Biogenesis,” Proceedings of the Nutrition Society 63 (2007): 279–286. 10.1079/PNS2004335 15294044

[23] M. Zhou , B.‐Z. Lin , S. Coughlin , G. Vallega , and P. F. Pilch , “UCP‐3 Expression in Skeletal Muscle: Effects of Exercise, Hypoxia, and AMP‐Activated Protein Kinase,” American Journal of Physiology. Endocrinology and Metabolism 279 (2000): E622–E629. 10950831 10.1152/ajpendo.2000.279.3.E622

[24] W. Tang , S. Tang , H. Wang , Z. Ge , D. Zhu , and Y. Bi , “Insulin Restores UCP3 Activity and Decreases Energy Surfeit to Alleviate Lipotoxicity in Skeletal Muscle,” International Journal of Molecular Medicine 40 (2017): 2000–2010. 29039450 10.3892/ijmm.2017.3169

[25] M. Ahsan , L. Garneau , and C. Aguer , “The Bidirectional Relationship Between AMPK Pathway Activation and Myokine Secretion in Skeletal Muscle: How It Affects Energy Metabolism,” Frontiers in Physiology 13 (2022): 1040809. 36479347 10.3389/fphys.2022.1040809 PMC9721351

[26] M. Waldeck‐Weiermair , R. Malli , S. Naghdi , M. Trenker , M. J. Kahn , and W. F. Graier , “The Contribution of UCP2 and UCP3 to Mitochondrial Ca^2+^ Uptake Is Differentially Determined by the Source of Supplied Ca^2+^ ,” Cell Calcium 47 (2010): 433–440. 20403634 10.1016/j.ceca.2010.03.004

[27] D. C. Wright , K. A. Hucker , J. O. Holloszy , and D. H. Han , “Ca^2+^ and AMPK Both Mediate Stimulation of Glucose Transport by Muscle Contractions,” Diabetes 53 (2004): 330–335. 14747282 10.2337/diabetes.53.2.330

[28] L. C. Ching , C. Y. Chen , K. H. Su , et al., “Implication of AMP‐Activated Protein Kinase in Transient Receptor Potential Vanilloid Type 1‐Mediated Activation of Endothelial Nitric Oxide Synthase,” Molecular Medicine 18 (2012): 805–815. 22451268 10.2119/molmed.2011.00461 PMC7751829

[29] R. Sartori , V. Romanello , and M. Sandri , “Mechanisms of Muscle Atrophy and Hypertrophy: Implications in Health and Disease,” Nature Communications 12 (2021): 330. 10.1038/s41467-020-20123-1 PMC7803748 33436614

[30] C. Rommel , B. A. Clarke , S. Zimmermann , et al., “Differentiation Stage‐Specific Inhibition of the Raf‐MEK‐ERK Pathway by Akt,” Science 286 (1999): 1738–1741. 10576741 10.1126/science.286.5445.1738

[31] D. J. Glass , “Molecular Mechanisms Modulating Muscle Mass,” Trends in Molecular Medicine 9 (2003): 344–350. 12928036 10.1016/s1471-4914(03)00138-2

[32] J. Wang , X. Yao , and J. Huang , “New Tricks for Human Farnesyltransferase Inhibitor: Cancer and Beyond,” MedChemComm 8 (2017): 841–854. 30108801 10.1039/c7md00030h PMC6072492

[33] F. Morgillo and H. Y. Lee , “Lonafarnib in Cancer Therapy,” Expert Opinion on Investigational Drugs 15 (2006): 709–719. 16732721 10.1517/13543784.15.6.709

[34] L. R. Smith , G. Meyer , and R. L. Lieber , “Systems Analysis of Biological Networks in Skeletal Muscle Function,” Wiley Interdisciplinary Reviews. Systems Biology and Medicine 5 (2013): 55–71. 23188744 10.1002/wsbm.1197 PMC4076960

[35] N. Yang , D. G. MacArthur , J. P. Gulbin , et al., “ACTN3 Genotype Is Associated With Human Elite Athletic Performance,” American Journal of Human Genetics 73 (2003): 627–631. 12879365 10.1086/377590 PMC1180686

[36] C. Handschin and B. M. Spiegelman , “The Role of Exercise and PGC1alpha in Inflammation and Chronic Disease,” Nature 454 (2008): 463–469. 18650917 10.1038/nature07206 PMC2587487

[37] C. Koh , L. Canini , H. Dahari , et al., “Oral Prenylation Inhibition With Lonafarnib in Chronic Hepatitis D Infection: A Proof‐of‐Concept Randomised, Double‐Blind, Placebo‐Controlled Phase 2A Trial,” Lancet Infectious Diseases 15 (2015): 1167–1174. 26189433 10.1016/S1473-3099(15)00074-2 PMC4700535

[38] J. R. Zierath and J. A. Hawley , “Skeletal Muscle Fiber Type: Influence on Contractile and Metabolic Properties,” PLoS Biology 2 (2004): e348. 15486583 10.1371/journal.pbio.0020348 PMC521732

[39] S. Mankhong , S. Kim , S. Moon , H. B. Kwak , D. H. Park , and J. H. Kang , “Experimental Models of Sarcopenia: Bridging Molecular Mechanism and Therapeutic Strategy,” Cells 9 (2020): 1385. 32498474 10.3390/cells9061385 PMC7348939

[40] L. A. Herndon , P. J. Schmeissner , J. M. Dudaronek , et al., “Stochastic and Genetic Factors Influence Tissue‐Specific Decline in Ageing C. Elegans,” Nature 419 (2002): 808–814. 12397350 10.1038/nature01135

