# Supplementary material for: Lonafarnib Protects Against Muscle Atrophy Induced by Dexamethasone
Source: J Cachexia Sarcopenia Muscle. 2024 Dec 17;16(1):e13665. doi: 10.1002/jcsm.13665 (PMC11696026; doi:10.1002/jcsm.13665)
Supplement: Supplementary file 1 — Table S1. Antibodies for the immunoassays. Table S2. Primers for the qRT–PCR analysis. Table S3. Summary of the sequencing statistics. Table S4. Summary of the biological processes affected by lonafarnib application. Figure S1. Analysis of the muscle‐to‐body weight ratio shows the ratio of muscle weight to total body weight in various muscles: gastrocnemius (a), tibialis anterior (b), soleus (c), and plantaris (d) muscles. Weights of specific hindlimb muscles— soleus (e), and plantaris (f)—across groups. *p < 0.05 by one‐way ANOVA with the Games–Howell post hoc test. CTL = control, CL1 = control that received 1 μM lonafarnib, CL5 = control that received 5 μM lonafarnib, DEX = dexamethasone‐induced sarcopenia model, DL1 = dexamethasone‐induced sarcopenia model that received 1 μM lonafarnib, DL5 = dexamethasone‐induced sarcopenia model that received 5 μM lonafarnib. Figure S2. Electrophysiological analysis of compound muscle action potentials. Quantitative results of the onset latency and duration of compound muscle action potentials in the gastrocnemius (a, b) and tibialis anterior (c, d) muscles across different groups: control (CTL), dexamethasone‐induced sarcopenia (DEX) models, and models treated with 1 or 5 μM lonafarnib (CL1, CL5, DL1, and DL5). Figure S3. Bioinformatics analysis of the RNA‐seq data and sample correlations. (a) The boxplot shows the gene expression in each sample after data normalization. The ordinate represents the gene expression value, and the abscissa represents the control, DEX, DL1, and DL5 groups in both GC and TA muscle with independent colours. The distribution of FPKM values for total expressed genes in the samples of each group is also shown. (b) The PCA plot shows how similar and close the transcriptome changes in each sample are based on the global gene expression level. (c) Heatmap clustering analysis of globally expressed genes is shown. The histogram in the colour key at the top shows the expression values. Figure S4. Statistica [file JCSM-16-e13665-s001.docx]

**Supplementary data**

**Supplementary Table 1. Antibodies for the immunoassays**

| Antibody | Host | Company | Catalog No. | Dilution |
| --- | --- | --- | --- | --- |
| MYH | Mouse | Santa Cruz | sc-376157 | 1:500 |
| Dystrophin | Mouse | Santa Cruz | sc-365954 | 1:500 |
| Myosin heavy chain  (slow, alpha- and beta-) | Mouse (IgG2b) | DSHB | BA-F8 | 1:50 |
| Myosin heavy chain Type IIA | Mouse (IgG1) | DSHB | SC-71 | 1:600 |
| Myosin heavy chain Type IIB | Mouse (IgM) | DSHB | BF-F3 | 1:100 |
| p-PTEN (Ser380) | Rabbit | Cell Signaling | #9551 | 1:500 |
| PTEN | Rabbit | Cell Signaling | #9552 | 1:500 |
| p-Akt (Ser473) | Rabbit | Cell Signaling | #9271 | 1:500 |
| p-Akt (Thr308) | Rabbit | Cell Signaling | #9275 | 1:500 |
| Akt | Rabbit | Cell Signaling | #9272 | 1:500 |
| p-mTOR (Ser2448) | Rabbit | Cell Signaling | #2971 | 1:500 |
| mTOR | Rabbit | Cell Signaling | #2972 | 1:500 |
| p-P70S6K (Thr389) | Rabbit | Cell Signaling | #9234 | 1:500 |
| P70S6K | Rabbit | Cell Signaling | #9202 | 1:500 |
| p-S6 Ribosomal Protein (Ser235/236) | Rabbit | Cell Signaling | #2211 | 1:500 |
| p-S6 Ribosomal Protein (Ser240/244) | Rabbit | Cell Signaling | #5364 | 1:500 |
| S6 Ribosomal Protein | Mouse | Cell Signaling | #2317 | 1:500 |
| FoxO1 | Rabbit | Cell Signaling | 2880S | 1:1000 |
| Fbx32 | Rabbit | Abcam | ab168372 | 1:500 |
| Mib1 | Mouse | Santa Cruz | sc-393551 | 1:1000 |
| Actn3 | Rabbit | Abcam | ab68204 | 1:2000 |
| Histone H3 | Rabbit | Invitrogen | PA5-16183 | 1:1000 |
| Angptl4 | Rabbit | Invitrogen | 40-9800 | 1:500 |
| PGC1-alpha | Rabbit | Invitrogen | PA5-72948 | 1:1000 |
| p-p44/42 MAPK (Erk1/2) (Thr202/Tyr204) | Rabbit | Cell Signaling | #4370 | 1:500 |
| p44/42 MAPK (Erk1/2) (137F5) | Rabbit | Cell Signaling | #4695 | 1:500 |
| GAPDH | Rabbit | Santa Cruz | SC-25778 | 1:1000 |
| β-actin | Mouse | Santa Cruz | SC-47778 | 1:1000 |
| p-AMPKa (Thr 172) | Rabbit | Cell Signaling | 2535 | 1:500 |
| AMPKa | Rabbit | Cell Signaling | 2603 | 1:500 |
| p-CamK2 (Thr 286) | Rabbit | Cell Signaling | 12716 | 1:500 |
| CamK2 (pan) | Rabbit | Cell Signaling | 4436 | 1:500 |
| H-Ras | Mouse | Santa Cruz | sc-29 | 1:100 |
| Cytochrome-C | Mouse | BD Pharmingen™ | 556432 | 1:200 |
| Alexa Fluor 350 IgG2b (blue) |  | Invitrogen | A21140 | 1:500 |
| Alexa Fluor 488 IgG1 (green) |  | Invitrogen | A21121 | 1:500 |
| Alexa Fluor 555 IgM (red) |  | Invitrogen | A21426 | 1:500 |
| anti-mouse, HRP |  | Jackson ImmunoResearch | 115-035-003 | 1:3000 |
| anti-rabbit, HRP |  | Jackson ImmunoResearch | 111-035-144 | 1:3000 |
|  |  |  |  |  |

**Supplementary Table 2. Primers for the qRT‒PCR analysis**

| Gene name |  | **Primer sequence** |
| --- | --- | --- |
| GAPDH | Forward | 5'-GGGAAGCCCATCACCATCT-3' |
|  | Reverse | 5'-CGGCCTCACCCCATTTG-3' |
| MuRF1 | Forward | 5'-TGTCTGGAGGTCGTTTCCG-3' |
|  | Reverse | 5'-GTGCCGGTCCATGATCACTT-3' |
| MAFbx | Forward | 5'-ATGCACACTGGTGCAGAGAG-3' |
|  | Reverse | 5'-TGTAAGCACACAGGCAGGTC-3' |
| UCP3 | Forward | 5'-CAACGGTTGTGAAGTTCCTG-3' |
|  | Reverse | 5'-CTCTGTGCGCACCATAGTCA-3' |
| ANGPLT4 | Forward | 5'-CTGGACAGTGATTCAGAGACGC-3' |
|  | Reverse | 5'-GATGCTGTGCATCTTTTCCAGGC-3' |

**Supplementary Table 3** **Summary of the sequencing statistics**

| **Tissues** | **Treatment** | **Total reads** | **Unique mapped reads (count)** | **%** | **Multiple mapped reads (count)** | **%** | **Unmapped reads (count)** | **%** |
| --- | --- | --- | --- | --- | --- | --- | --- | --- |
| **Tibialis anterior muscle (TA)** | DEX-1 | 48,186,414 | 44,244,845 | 91.82 | 3,081,614 | 6.40 | 859,955 | 1.78 |
|  | DEX-2 | 41,862,032 | 38,147,900 | 91.13 | 2,575,196 | 6.15 | 1,138,936 | 2.72 |
|  | DEX-4 | 42,052,421 | 38,630,763 | 91.86 | 2,478,909 | 5.89 | 942,749 | 2.24 |
|  | DEX_1 µM-1 | 45,059,558 | 41,336,417 | 91.74 | 2,874,326 | 6.38 | 848,815 | 1.88 |
|  | DEX_1 µM-2 | 41,277,504 | 37,847,615 | 91.69 | 2,700,839 | 6.54 | 729,050 | 1.77 |
|  | DEX_1 µM-4 | 45,926,221 | 42,205,007 | 91.90 | 2,946,657 | 6.42 | 774,557 | 1.69 |
|  | DEX_5 µM-1 | 47,509,126 | 43,502,930 | 91.57 | 3,154,280 | 6.64 | 851,916 | 1.79 |
|  | DEX_5 µM-2 | 57,334,477 | 52,572,988 | 91.70 | 3,760,353 | 6.56 | 1,001,136 | 1.75 |
|  | DEX_5 µM-4 | 38,147,710 | 34,481,102 | 90.39 | 2,770,346 | 7.26 | 896,262 | 2.35 |
|  | Wild-1 | 50,974,942 | 46,637,316 | 91.49 | 3,324,431 | 6.52 | 1,013,195 | 1.99 |
|  | Wild-2 | 52,338,227 | 47,983,770 | 91.68 | 3,497,630 | 6.68 | 856,827 | 1.64 |
|  | Wild-4 | 45,760,282 | 41,917,344 | 91.60 | 3,068,589 | 6.71 | 774,349 | 1.69 |
| **Gastrocnemius muscle (GC)** | DEX-1 | 48,063,312 | 44,357,606 | 92.29 | 2,869,718 | 5.97 | 835,988 | 1.74 |
|  | DEX-2 | 41,935,231 | 38,043,151 | 90.72 | 2,938,073 | 7.01 | 954,007 | 2.27 |
|  | DEX-4 | 47,147,324 | 43,471,803 | 92.20 | 2,799,564 | 5.94 | 875,957 | 1.86 |
|  | DEX_1 µM-1 | 45,609,448 | 41,930,427 | 91.93 | 2,814,111 | 6.17 | 864,910 | 1.90 |
|  | DEX_1 µM-2 | 47,341,892 | 43,649,590 | 92.20 | 2,782,482 | 5.88 | 909,820 | 1.92 |
|  | DEX_1 µM-4 | 44,907,130 | 41,322,829 | 92.02 | 2,730,662 | 6.08 | 853,639 | 1.90 |
|  | DEX_5 µM-1 | 42,986,806 | 39,617,350 | 92.16 | 2,597,771 | 6.04 | 771,685 | 1.80 |
|  | DEX_5 µM-2 | 48,370,885 | 44,388,617 | 91.77 | 3,122,780 | 6.46 | 859,488 | 1.78 |
|  | DEX_5 µM-4 | 52,060,553 | 47,624,672 | 91.48 | 3,501,918 | 6.73 | 933,963 | 1.79 |
|  | Wild-1 | 47,739,966 | 43,777,281 | 91.70 | 3,091,443 | 6.48 | 871,242 | 1.82 |
|  | Wild-2 | 45,837,034 | 42,046,186 | 91.73 | 2,849,887 | 6.22 | 940,961 | 2.05 |
|  | Wild-4 | 48,536,306 | 44,586,929 | 91.86 | 3,138,548 | 6.47 | 810,829 | 1.67 |

**Supplementary Table 4. Summary of the biological processes affected by lonafarnib application**

| **Term id** | **Term name** | **Description** | **Intersection size** | **p value** | **Precision** | **Query size** | **Recall** | **Source** | **Term size** |
| --- | --- | --- | --- | --- | --- | --- | --- | --- | --- |
| GO:0030168 | Platelet activation | "A series of progressive, overlapping events triggered by exposure of the platelets to subendothelial tissue. These events include shape change, adhesiveness, aggregation, and release reactions. When carried through to completion, these events lead to the formation of a stable hemostatic plug." [http://www.graylab.ac.uk/omd/] | 9 | 6.98E-08 | 0.173076923 | 52 | 0.067669173 | Gene Ontology Biological Process | 133 |
| GO:1903305 | Regulation of regulated secretory pathway | "Any process that modulates the frequency, rate or extent of regulated secretory pathway." [GO_REF:0000058, GOC:pad, GOC:PARL, GOC:TermGenie, PMID:12526776] | 6 | 0.001414337 | 0.115384615 | 52 | 0.046875 | Gene Ontology Biological Process | 128 |
| GO:0051896 | Regulation of phosphatidylinositol 3-kinase | "Any process that modulates the frequency, rate or extent of phosphatidylinositol 3-kinase/protein kinase B signal transduction. Source: GOC:ai" | 7 | 1.42E-07 | 0.076923077 | 52 | 0.0175 | Gene Ontology Biological Process | 101 |
| GO:0061337 | Cardiac conduction | "Transfer of an organized electrical impulse across the heart to coordinate the contraction of cardiac muscles. The process begins with generation of an action potential (in the sinoatrial node (SA) in humans) and ends with a change in the rate, frequency, or extent of the contraction of the heart muscles." [GOC:dph] | 8 | 1.73E-07 | 0.153846154 | 52 | 0.083333333 | Gene Ontology Biological Process | 96 |
| GO:0150104 | Transport across blood‒brain barrier | "The directed movement of substances (e.g., macromolecules, small molecules, ions) through the blood‒brain barrier." [GOC:aruk, GOC:bc, PMID:29377008] | 10 | 2.30E-11 | 0.192307692 | 52 | 0.114942529 | Gene Ontology Biological Process | 87 |
| GO:0050848 | Regulation of calcium-mediated signaling | "Any process that modulates the frequency, rate or extent of calcium-mediated signaling, the process in which a cell uses calcium ions to convert an extracellular signal into a response." [GOC:ai] | 5 | 0.003259031 | 0.096153846 | 52 | 0.061728395 | Gene Ontology Biological Process | 81 |
| GO:0051148 | Negative regulation of muscle cell differentiation | "Any process that stops, prevents, or reduces the frequency, rate or extent of muscle cell differentiation." [CL:0000187, GOC:ai] | 6 | 1.48E-05 | 0.115384615 | 52 | 0.1 | Gene Ontology Biological Process | 60 |
| GO:0070509 | Calcium ion import | "The directed movement of calcium ions into a cell or organelle." [GOC:mah] | 6 | 3.72E-06 | 0.115384615 | 52 | 0.125 | Gene Ontology Biological Process | 48 |
| GO:0032922 | Circadian regulation of gene expression | "Any process that modulates the frequency, rate or extent of gene expression such that an expression pattern recurs with a regularity of approximately 24 hours." [GOC:mah] | 8 | 1.29E-08 | 0.153846154 | 52 | 0.114285714 | Gene Ontology Biological Process | 70 |
| GO:0042310 | Vasoconstriction | "A decrease in the diameter of blood vessels, especially arteries, due to constriction of smooth muscle cells that line the vessels, and usually causing an increase in blood pressure." [GOC:pr, ISBN:0192800752] | 6 | 9.96E-05 | 0.115384615 | 52 | 0.073170732 | Gene Ontology Biological Process | 82 |
| GO:1900121 | Negative regulation of receptor binding | "Any process that stops, prevents or reduces the frequency, rate or extent of a protein or other molecule binding to a receptor." [GOC:signaling, GOC:TermGenie] | 4 | 2.09E-05 | 0.076923077 | 52 | 0.363636364 | Gene Ontology Biological Process | 11 |


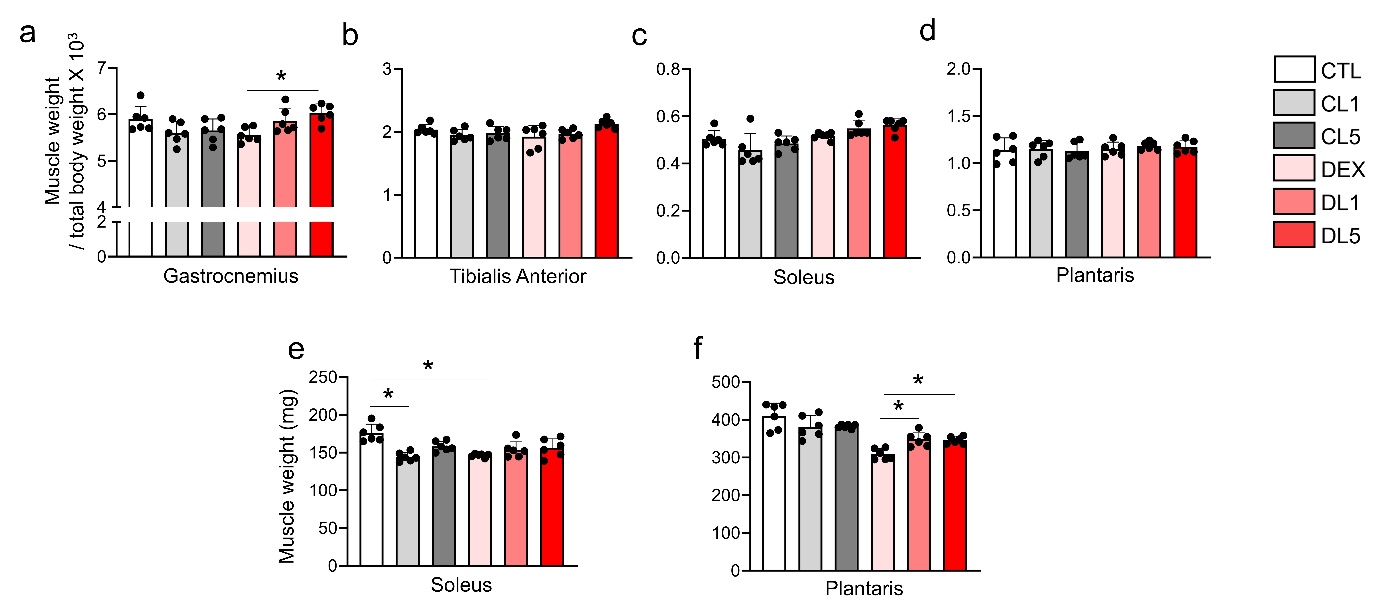


**Supplementary Figure 1. Analysis of the muscle-to-body weight ratio shows the ratio of muscle weight to total body weight in various muscles: gastrocnemius (a), tibialis anterior (b), soleus (c), and plantaris (d) muscles.** Weights of specific hindlimb muscles— soleus (e), and plantaris (f)—across groups. *p < 0.05 by one-way ANOVA with the Games–Howell post hoc test. CTL = control, CL1 = control that received 1 μM lonafarnib, CL5 = control that received 5 μM lonafarnib, DEX = dexamethasone-induced sarcopenia model, DL1 = dexamethasone-induced sarcopenia model that received 1 μM lonafarnib, DL5 = dexamethasone-induced sarcopenia model that received 5 μM lonafarnib.


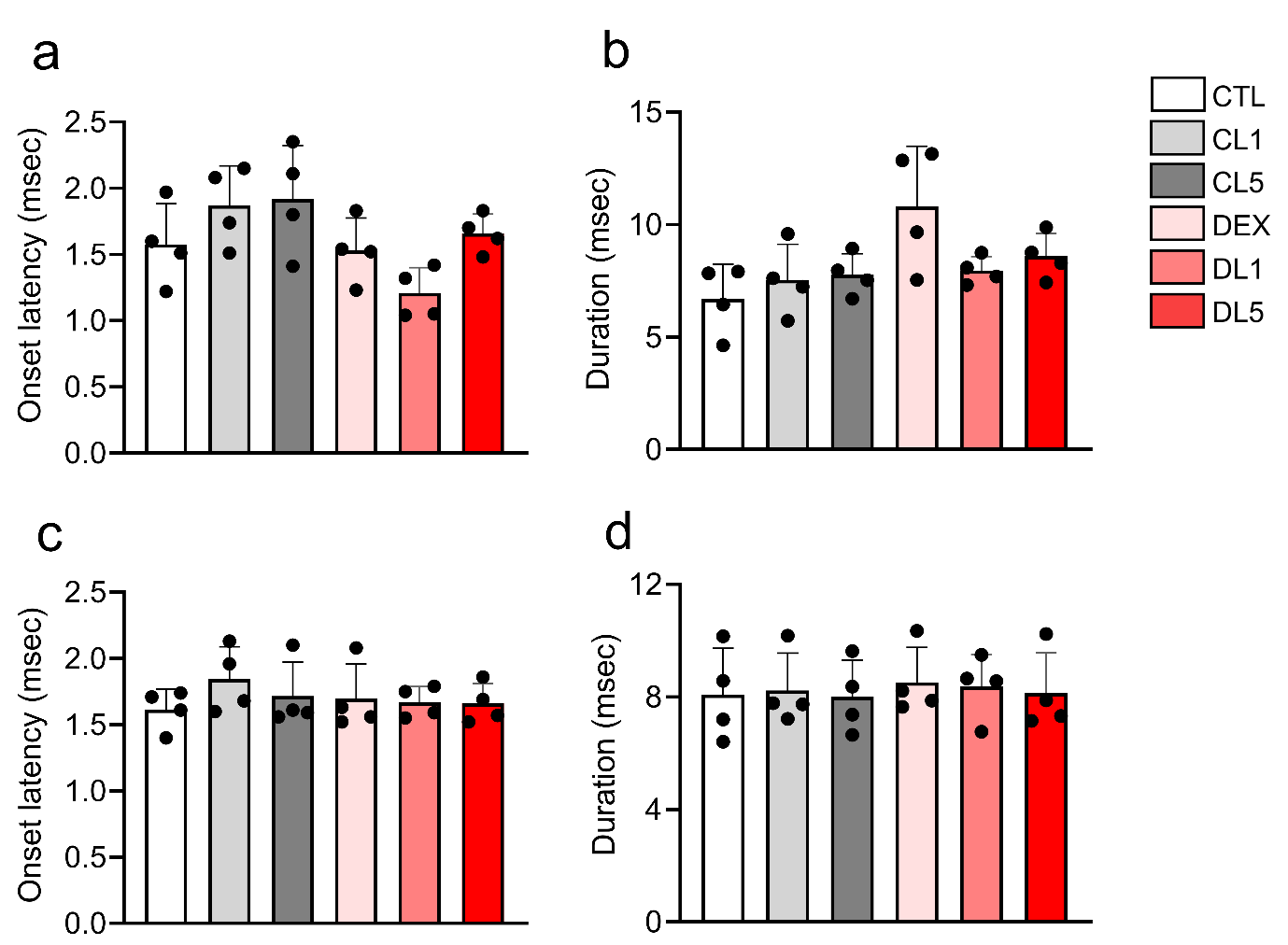


**Supplementary Figure 2. Electrophysiological analysis of compound muscle action potentials.** Quantitative results of the onset latency and duration of compound muscle action potentials in the gastrocnemius (a, b) and tibialis anterior (c, d) muscles across different groups: control (CTL), dexamethasone-induced sarcopenia (DEX) models, and models treated with 1 or 5 μM lonafarnib (CL1, CL5, DL1, and DL5).

**
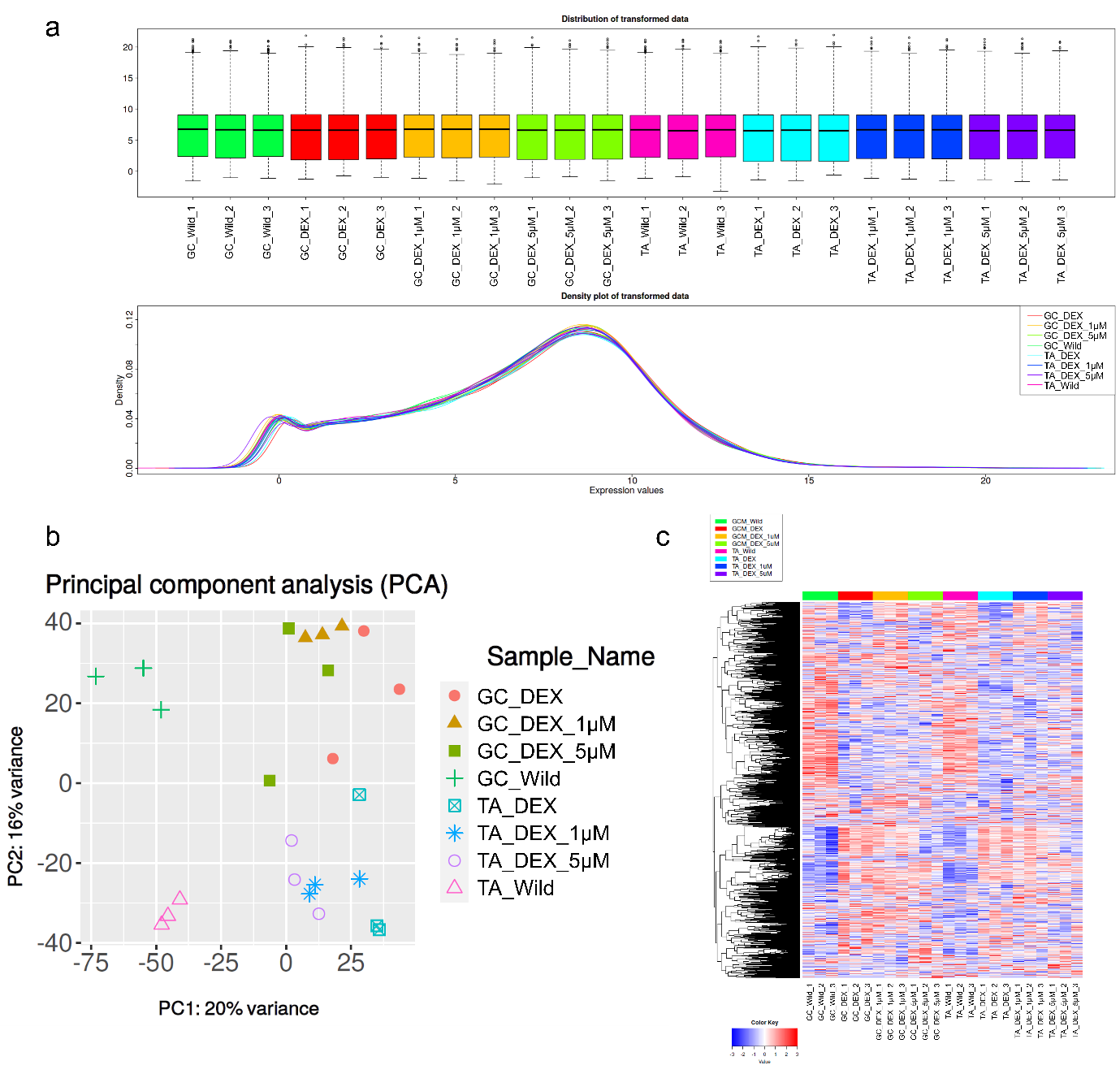
**

**Supplementary Figure 3. Bioinformatics analysis of the RNA-seq data and sample correlations.** (a) The boxplot shows the gene expression in each sample after data normalization. The ordinate represents the gene expression value, and the abscissa represents the control, DEX, DL1, and DL5 groups in both GC and TA muscle with independent colors. The distribution of FPKM values for total expressed genes in the samples of each group is also shown. (b) The PCA plot shows how similar and close the transcriptome changes in each sample are based on the global gene expression level. (c) Heatmap clustering analysis of globally expressed genes is shown. The histogram in the color key at the top shows the expression values.

**
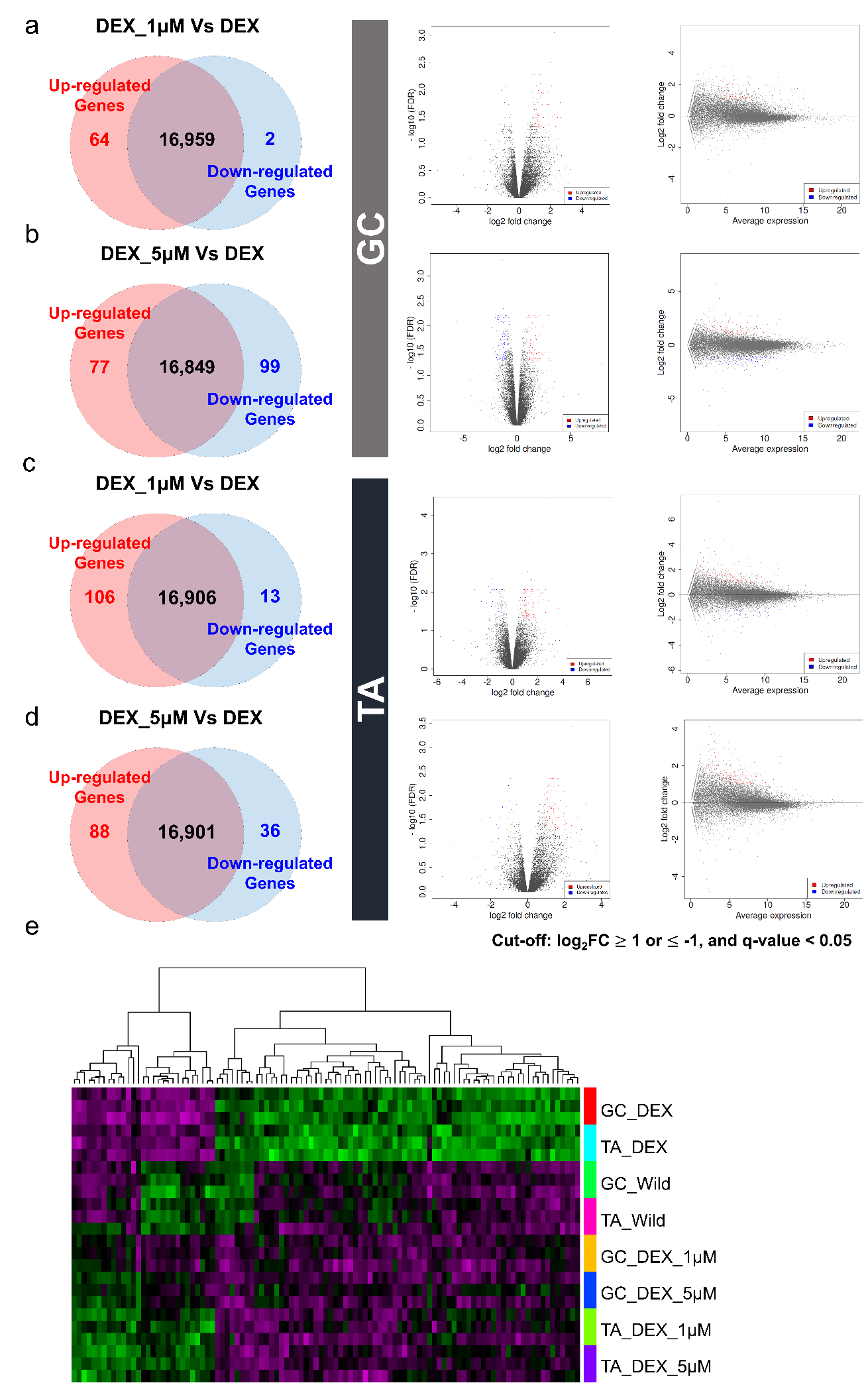
**

**Supplementary Figure 4. Statistical comparison of DEGs in both tissues between the DEX group and the DEX group.** The numbers of up- and downregulated genes identified in the four comparison sets are shown: (a) GC-DL1, (b) GC-DL5, (c) TA-DL1, and (d) TA-DL5. The FPKM volcano plot and MA plot were constructed by pairwise comparison against DEX gene expression. In this statistical analysis, red and blue dots represent the statistically significant up- and downregulated DEGs, respectively. The middle line with black dots indicates no difference in the mean expression values between samples. (e) Hierarchical clustering analysis of genes commonly identified in at least three comparison groups in the comparison of DEG and DEX groups for each tissue.**
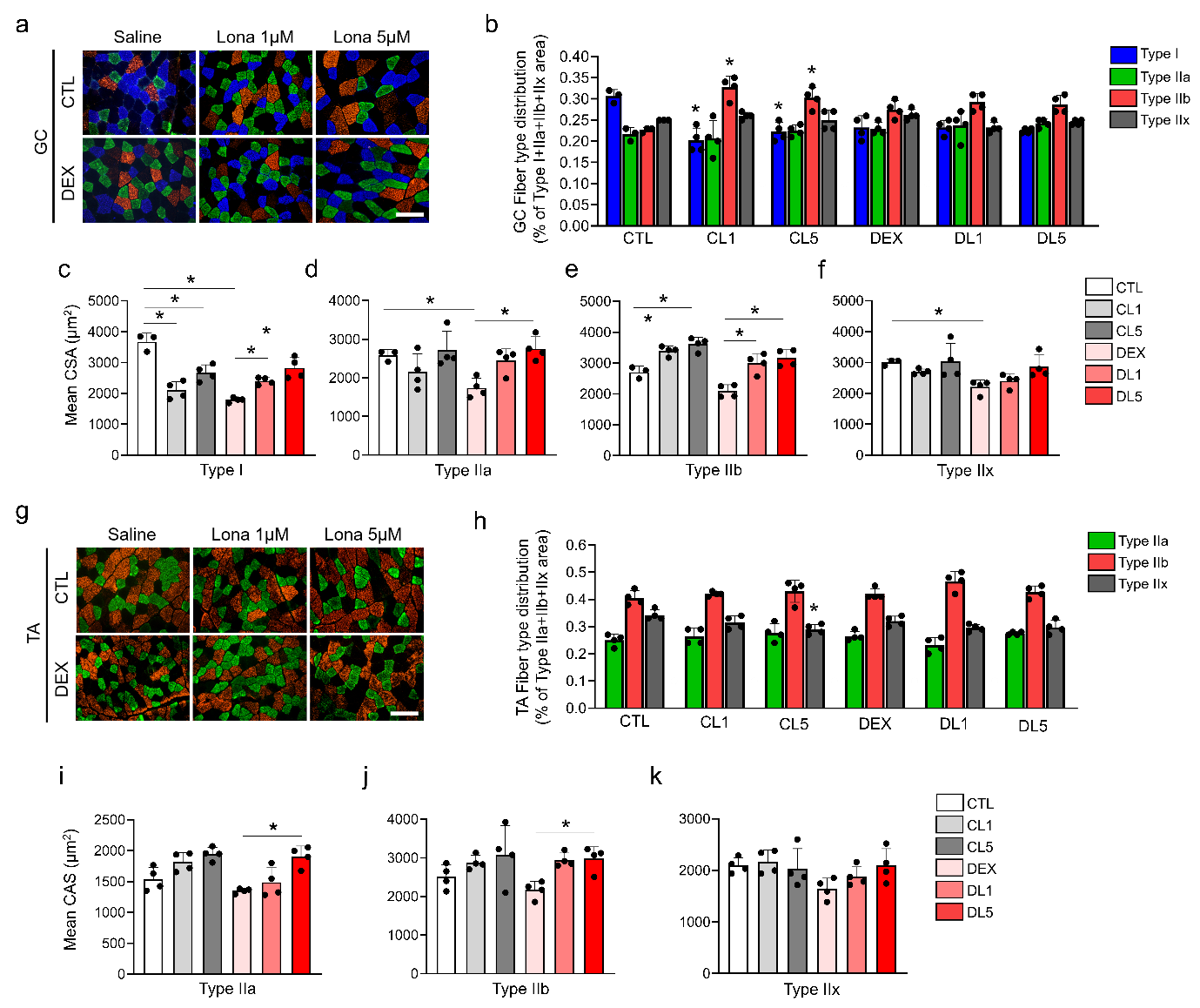
**

**Supplementary Figure 5. Effect of lonafarnib on muscle fiber type and gene expression.** Representative images (a) and quantitative analysis of cross-sectional areas of type I (blue), type IIa (green), type IIb (red), and type X (black) fibers in the gastrocnemius muscle in each group (b) and myotube areas of type I (c), type IIa (d), type IIb (e), and type X (f) fibers in the gastrocnemius muscle in each group. Representative images (g) and quantitative analysis of cross-sectional areas of type IIa (green) and type IIb (red) fibers in the tibialis anterior muscle (h) and myotube areas of type I (i), type IIa (j), and type IIb (k) fibers in the tibialis anterior muscle in each group. *p < 0.05 compared to the control (CTL) or sarcopenia (DEX) groups by one-way ANOVA with the Games-Howell post hoc test.

**
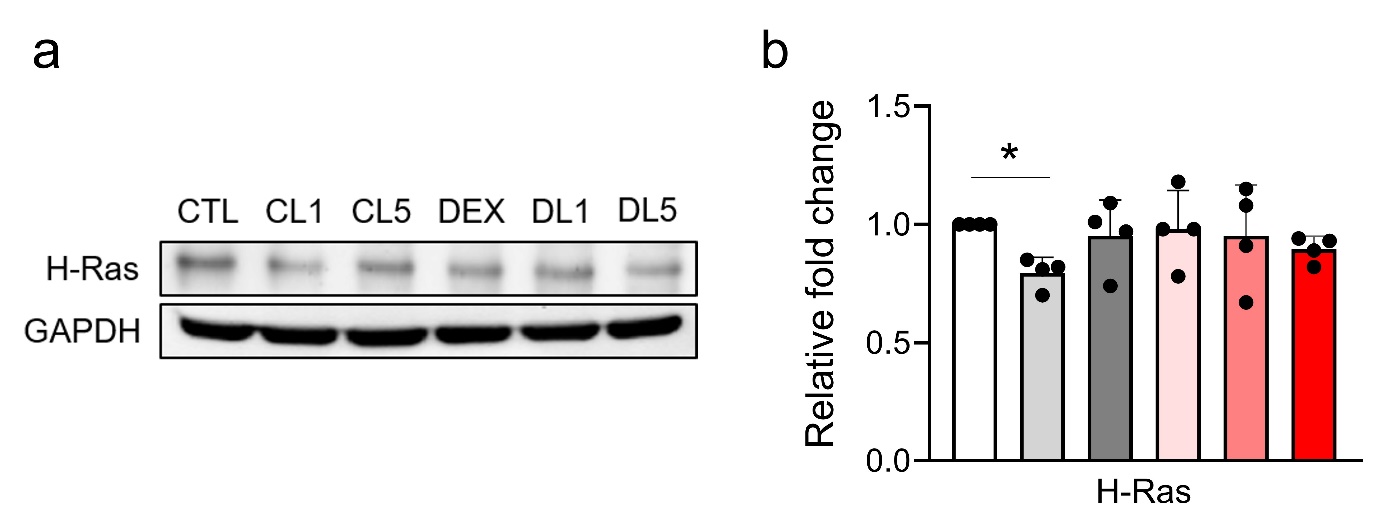
**

**Supplementary Figure 6. Western blot analysis of key genes in sarcopenia models after treatment with lonafarnib.** Representative images of western blots (a) and quantitative analysis of H-Ras (b). *p < 0.05 by one-way ANOVA with the Games–Howell post hoc test.

**
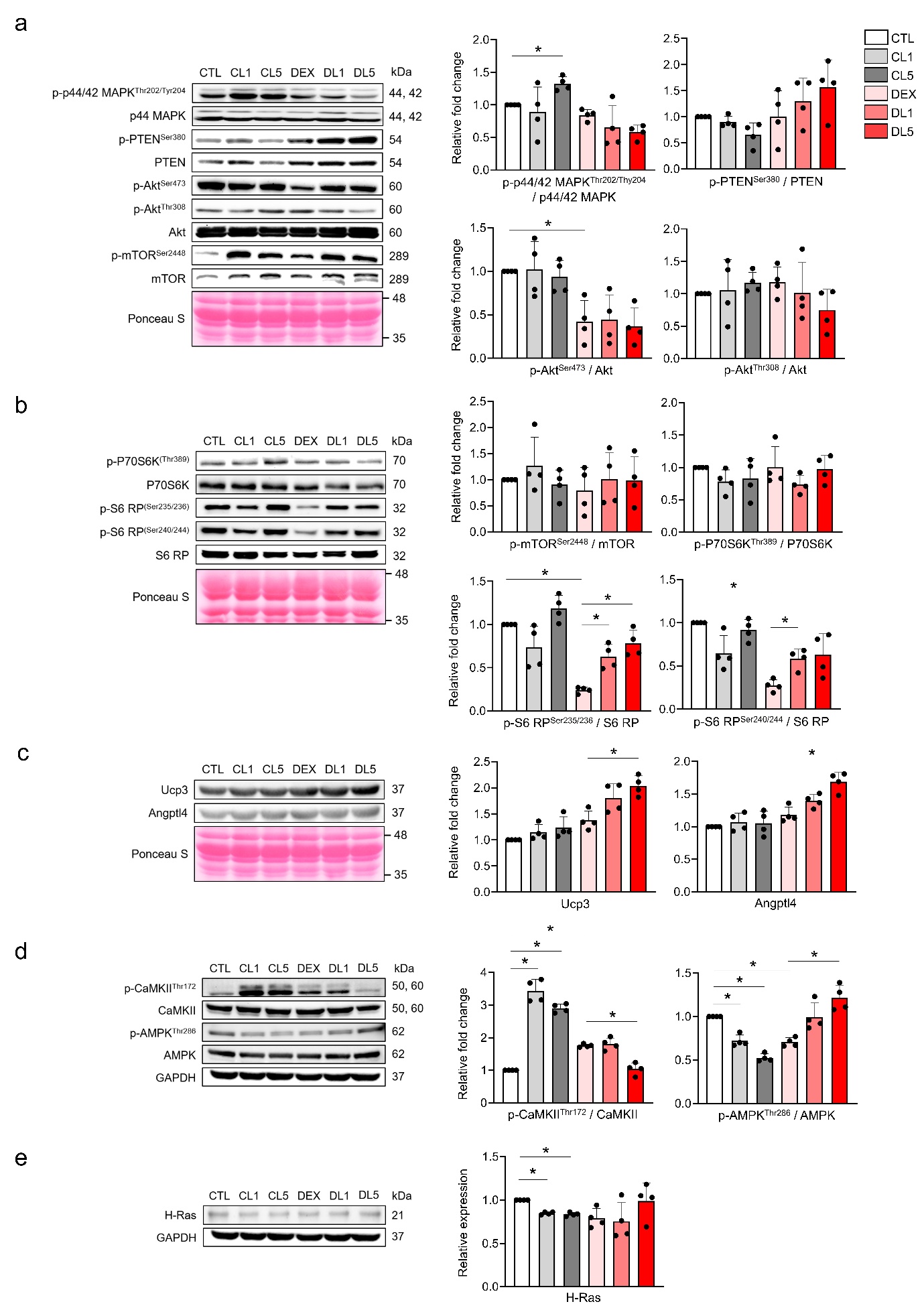
**

**Supplementary Figure 7. Western blot analysis of key genes in sarcopenia models after treatment with lonafarnib.** (a) Representative images of western blots (left) and quantitative analysis of the phosphorylated p44/42^Thr202/Thy204^(p-p44/42^Thr202/Thy204^)-to-p44/42 MAPK ratio, the phosphorylated PTEN^Ser380^ (p-PTEN^Ser380^)-to-PTEN ratio, the p-Akt^Ser473^-to-Akt ratio, p-Akt^Thr308^-to-Akt ratio and the p-mTOR^Ser2448^-to-mTOR ratio (right). (b) Representative western blot images (left) and quantitative analysis of the phosphorylated P70S6K^Thr389^/P70S6K ratio, the phosphorylated S6 RP^Ser235/236^/S6 RP ratio, and the phosphorylated S6 RP^Ser240/244^/S6 RP ratio (right), (c) representative western blot images (left) and quantitative analysis of Ucp3 and Angptl4 expression (right), (d) representative western blot images (left) and quantitative analysis of the phosphorylated CaMKII^Thr172^/CaMKII ratio (p-CaMKII/CaMKII) and phosphorylated AMPK^Thr286^/AMPK ratio (p-AMPK/AMPK) (right), and (e) representative images of western blots (left) and quantitative analysis of H-Ras (right). *p < 0.05 by one-way ANOVA with the Games–Howell post hoc test.

Supplementary References

S1. Beaudart C, Zaaria M, Pasleau F, Reginster J-Y, Bruyère O. Health Outcomes of Sarcopenia: A Systematic Review and Meta-Analysis. Plos One. 2017;12: e0169548.

S2. Marzetti E, Calvani R, Tosato M, Cesari M, Di Bari M, Cherubini A, et al. Sarcopenia: an overview. Aging Clin Exp Res. 2017;29:11-7.

S3. Sandri M, Sandri C, Gilbert A, Skurk C, Calabria E, Picard A, et al. Foxo transcription factors induce the atrophy-related ubiquitin ligase atrogin-1 and cause skeletal muscle atrophy. Cell. 2004;117:399-412.

S4. Schakman O, Kalista S, Barbe C, Loumaye A, Thissen JP. Glucocorticoid-induced skeletal muscle atrophy. Int J Biochem Cell Biol. 2013;45:2163-72.

S5. Parahiba SM, Ribeiro ECT, Correa C, Bieger P, Perry IS, Souza GC. Effect of testosterone supplementation on sarcopenic components in middle-aged and elderly men: A systematic review and meta-analysis. Exp Gerontol. 2020;142:111106.

S6. Brioche T, Kireev RA, Cuesta S, Gratas-Delamarche A, Tresguerres JA, Gomez-Cabrera MC, et al. Growth hormone replacement therapy prevents sarcopenia by a dual mechanism: improvement of protein balance and of antioxidant defenses. J Gerontol A Biol Sci Med Sci. 2014;69:1186-98.

S7. Fonseca G, Dworatzek E, Ebner N, Von Haehling S. Selective androgen receptor modulators (SARMs) as pharmacological treatment for muscle wasting in ongoing clinical trials. Expert Opin Investig Drugs. 2020;29:881-91.

S8. group Ls, Achison M, Adamson S, Akpan A, Aspray T, Avenell A, et al. Effect of perindopril or leucine on physical performance in older people with sarcopenia: the LACE randomized controlled trial. J Cachexia Sarcopenia Muscle. 2022;13:858-71.

S9. Lincoff AM, Bhasin S, Flevaris P, Mitchell LM, Basaria S, Boden WE, et al. Cardiovascular Safety of Testosterone-Replacement Therapy. New England Journal of Medicine. 2023;389:107-17.

S10. Sepp-Lorenzino L, Ma Z, Rands E, Kohl NE, Gibbs JB, Oliff A, et al. A peptidomimetic inhibitor of farnesyl:protein transferase blocks the anchorage-dependent and -independent growth of human tumor cell lines. Cancer Res. 1995;55:5302-9.

S11. Capell BC, Erdos MR, Madigan JP, Fiordalisi JJ, Varga R, Conneely KN, et al. Inhibiting farnesylation of progerin prevents the characteristic nuclear blebbing of Hutchinson-Gilford progeria syndrome. Proc Natl Acad Sci U S A. 2005;102:12879-84.

S12. Yang SH, Bergo MO, Toth JI, Qiao X, Hu Y, Sandoval S, et al. Blocking protein farnesyltransferase improves nuclear blebbing in mouse fibroblasts with a targeted Hutchinson-Gilford progeria syndrome mutation. Proc Natl Acad Sci U S A. 2005;102:10291-6.

S13. Dhillon S. Lonafarnib: First Approval. Drugs. 2021;81:283-9.

S14. Hernandez I, Luna G, Rauch JN, Reis SA, Giroux M, Karch CM, et al. A farnesyltransferase inhibitor activates lysosomes and reduces tau pathology in mice with tauopathy. Sci Transl Med. 2019;11:eaat3005.

S15. Nakazawa H, Yamada M, Tanaka T, Kramer J, Yu YM, Fischman AJ, et al. Role of protein farnesylation in burn-induced metabolic derangements and insulin resistance in mouse skeletal muscle. PLoS One. 2015;10:e0116633.

S16. Rommel C, Bodine SC, Clarke BA, Rossman R, Nunez L, Stitt TN, et al. Mediation of IGF-1-induced skeletal myotube hypertrophy by PI(3)K/Akt/mTOR and PI(3)K/Akt/GSK3 pathways. Nat Cell Biol. 2001;3:1009-13.

S17. Mendoza MC, Er EE, Blenis J. The Ras-ERK and PI3K-mTOR pathways: cross-talk and compensation. Trends Biochem Sci. 2011;36:320-8.

S18. Powers SK, Morton AB, Hyatt H, Hinkley MJ. The Renin-Angiotensin System and Skeletal Muscle. Exerc Sport Sci Rev. 2018;46:205-14.

S19. Avruch J, Khokhlatchev A, Kyriakis JM, Luo Z, Tzivion G, Vavvas D, et al. Ras activation of the Raf kinase: tyrosine kinase recruitment of the MAP kinase cascade. Recent Prog Horm Res. 2001;56:127-55.

S20. Murphy DP, Nicholson T, Jones SW, O'Leary MF. MyoCount: a software tool for the automated quantification of myotube surface area and nuclear fusion index. Wellcome Open Res. 2019;4:6.

S21. Chow DK, Glenn CF, Johnston JL, Goldberg IG, Wolkow CA. Sarcopenia in the Caenorhabditis elegans pharynx correlates with muscle contraction rate over lifespan. Exp Gerontol. 2006;41:252-60.

S22. Huang C, Xiong C, Kornfeld K. Measurements of age-related changes of physiological processes that predict lifespan of Caenorhabditis elegans. Proc Natl Acad Sci U S A. 2004;101:8084-9.

S23. Prabakaran AD, McFarland K, Miz K, Durumutla HB, Piczer K, El Abdellaoui Soussi F, et al. Intermittent glucocorticoid treatment improves muscle metabolism via the PGC1alpha/Lipin1 axis in an aging-related sarcopenia model. J Clin Invest. 2024;134:e177427.

S24. Lakens D. Equivalence Tests: A Practical Primer for t Tests, Correlations, and Meta-Analyses, Soc Psychol Personal Sci. 2017; 8: 355-362.

S25. Mo X, Shen L, Cheng R, Wang P, Wen L, Sun Y, et al. Faecal microbiota transplantation from young rats attenuates age-related sarcopenia revealed by multiomics analysis. J Cachexia Sarcopenia Muscle. 2023;14:2168-83.

S26. Zangarelli A, Chanseaume E, Morio B, Brugere C, Mosoni L, Rousset P, et al. Synergistic effects of caloric restriction with maintained protein intake on skeletal muscle performance in 21-month-old rats: a mitochondria-mediated pathway. FASEB J. 2006;20:2439-50.
